# Biosensing applications of gold-functionalized carbon-based composites

**DOI:** 10.1007/s00449-026-03318-6

**Published:** 2026-03-26

**Authors:** Timothy O. Ajiboye, Abolaji A. Mafolasire, Idris O. Sanusi, Lawrence Sawunyama, Joshua Oyetade, Sheriff A. Balogun, Lebea N. Nthunya, Subhendu Dhibar, Agnes Pholosi

**Affiliations:** 1 Material Science Innovation and Modelling (MaSIM) Research Focus Area, Faculty of Natural and Agricultural Sciences, North-West University, Mafikeng Campus, Private Bag X2046, Mmabatho, 2735 South Africa; 2 Department of Chemistry, University of Ibadan, Ibadan, 200284 Nigeria; 3 Department of Pharmaceutical Chemistry and Analysis, Kampala International University, Western-Campus, Ishaka, Bushenyi, Uganda; 4 Department of Chemistry, School of Chemical and Physical Sciences, Faculty of Natural and Agricultural Sciences, North-West University, Mafikeng Campus, Private bag X2046, Mmabatho, 2735 South Africa; 5 Department of Chemistry, School of Physical and Mineral Sciences, University of Limpopo (Turfloop), Polokwane, 0727 Sovenga South Africa; 6 Institute for Nanotechnology and Water Sustainability, College of Science, Engineering and Technology, University of South Africa, Johannesburg, FL South Africa; 7 Department of Physics, Indian Institute of Technology Patna, Patna, Bihar 801106 India; 8 Department of Biotechnology and Chemistry, Vaal University of Technology, Private Bag X021, Vanderbijlpark, 1900 South Africa

**Keywords:** Gold, Carbon, Sensor, Biosensor, Nanoparticles

## Abstract

Gold nanoparticles (AuNPs) have been utilized for sensing purposes and various applications. Some of the challenges associated with the use of pristine gold nanoparticles for sensing application are oxidation and rapid aggregation. Functionalization with various organic and inorganic materials have been used to overcome these challenges. Examples of such organic materials are the carbon-based materials such as activated carbon, graphene, CNT, graphitic carbon nitride, cyclodextrin, fullerenes, gels, polymers, alginates, chitosan, biochar, coal, resins/wood, metal organic framework, graphene oxide/reduced graphene oxide and cellulose. The present review gives detailed overview of the biosensing applications of gold nanoparticles functionalized with various carbon-based materials. Various methods of biosensing such as electrochemical (amperometry, potentiometric, voltametric and impedimetric) as well as mass-based biosensing methods, are comprehensively discussed. Then, the use of the gold/carbon-based materials in detecting oligonucleotides/DNA, tumour cells, protein/enzymes, glucose, thiol, peroxide and microbes such as bacteria and viruses are comprehensively reviewed.

## Introduction

Gold nanoparticles (AuNPs) are made up of colloidal gold particles with sizes in nanometer range, prepared from the reduction of gold salts in the presence of capping and reducing agents. AuNPs can be synthesised in different sizes and shapes, such as nanospheres, nanoshells, nanoclusters, nanocubes, nanocages, branched and nanorods [1]. When gold nanoparticle interacts with light, the electrons on their surfaces result in plasmonic resonance, causing them to display a wide range of vibrant colours ranging from red to purple, depending on the size and shape [2]. Large sizes AuNPs display brown, purple, or blue colours, which progressively change to orange/red as the particle size reduces [3]. The shapes can be functionalized via different techniques to create the desired functionalities with different properties for various applications. Gold nanoparticles have been widely explored due to their unique chemical, electrical, catalytic and optical characteristics. They present superior characteristics such as low toxicity, high surface area, high stability against oxidation, high thermal conductivity, magnetic properties and excellent biocompatibility [4–7]. Furthermore, they have physico-chemical inertness, good conductors of electricity and heat, and easy to synthesize and characterize. Due to these unique characteristics, AuNPs are the most promising nanomaterials in electronics, therapeutics, biological sensors, biomedical, cancer diagnostics, catalysis, water remediation and nanomedicine.

AuNPs have exceptionally high absorption coefficients, allowing higher sensitivity in optical detection methods than conventional dyes. In biosensing, gold nanoparticles can be designed to selectively attach to certain biomarkers and improve their ability to detect a range of biological materials [8]. In biological applications, they are widely explored in drug delivery, diagnostics, bioimaging, sensing, and ultrasensitive detection [5]. The high chemical stability and the distinctive optical and electrochemical properties of gold nanoparticles, which depend on their size and shape, make them excellent options for use in photothermal therapy and targeted drug delivery. AuNPs’ strong surface plasmonic resonance (SPR) in the visible region has enabled them to be applied as colometry agents for sensing and detection of water pollutants [9]. On the other hand, their small size, resulting in a large surface area to volume ratio, allows them to interact with molecules, making them find great application in chemical sensing platforms and in the removal of pollutants from aqueous solution and wastewater [9]. Table 1 reveals the properties of AuNPs and their application.

**Table 1:** Properties of AuNPs and their application

Properties	Application
Biocompatibility, large surface area	Electrochemical biosensor, biomedical applications, and drug delivery
Redox activity	Electronic device and electrochemical sensing
Florescence quenching	Material Engineering and sensors development
Surface enhanced Raman scattering (SERS).	Imaging and sensing
Localized surface plasmonic resonance (LSPR)	Biosensing, Medical imaging, colorimetric sensing
	Catalysis, Adsorption

High surface-to-volume ratio, high stability, ease of surface modification, and biocompatibility

Various applications were often carried out by using pristine gold nanoparticles. One significant limitation in using pristine gold nanoparticles is agglomeration, which results from direct interactions between particles through Van der Waals forces and magnetic interactions. Agglomeration affects the particle size of the nanoparticles, leading to a substantial effect on the potential nanotoxicity and a significant change in the biological impact of the nanoparticles. Researchers have been exerting significant effort into stabilizing nanomaterials by modifying the nanoparticle surface or coordinating with ligands/anionic species. Stabilizers create repulsive forces that decisively counteract the attractive van der Waals forces, thereby preventing particle aggregation in a solution. Hence, biocompatible AuNPs with high surface-to-volume ratio and high surface reactivity have been synthesized using chemical and biological stabilizers as reducing agents.

A process aimed at enhancing the properties of gold nanoparticles is to form a composite with different carbon-based materials. Some of the carbon-based materials are activated carbon, graphene, CNT, graphitic carbon nitride, cyclodextrin, fullerenes, gels, polymers, alginates, chitosan, biochar, coal, resins/wood), metal organic framework, graphene oxide/reduced graphene oxide and cellulose [10]. These carbon-based materials are particularly advantageous as a composite material since they have strong mechanical strength, good thermal stability, high chemical stability, good electrical conductivity and large surface area [11]. These characteristics make them a suitable support material for gold nanoparticles. Their combination with gold enhances the reactivity and durability which are desirable characteristic for the nanocomposites. The functionalized gold nanoparticles show good adaptability and performance thereby enhancing their usage in several fields [12].

Despite the positive attributes of both gold nanoparticles and carbon-based materials, their synergistic potential as biosensor has not been reviewed. For instance, Li et al., [13] just reviewed biosensing application of selected materials (carbon nanotubes (CNTs), quantum dots (QDs), graphene, gold and magnetic nanobeads) [13]. Also, Mehrotra [14] discussed the application of various materials as piezoelectric biosensor, thermal biosensor, DNA biosensors, tissue-based biosensors and enzyme-based biosensors. The reports did not focus on gold composite and did not discuss peroxide detection. Considering these gaps, the present review focuses on the utilization of the composite made from these carbon-based materials and gold for detecting substances in the biological system.

## Methods of biosensing

### Electrochemical biosensors

Electrochemical biosensors (ECB) are a widely explored and extensively applied class of biosensors that harness electrochemical transduction principles to detect and quantify biological analytes [15–18]. They operate by converting a biochemical interaction, typically between a biological recognition element and a target analyte, into an electrical signal that can be measured and interpreted. The biological recognition element might include enzymes, antibodies, nucleic acids, or cells, while the transducer measures parameters like current (amperometric sensors), potential (potentiometric sensors), or impedance (impedimetric sensors) [19]. Their advantage lies in their simplicity, cost-effectiveness, high sensitivity, and capacity for miniaturization, making them ideal for point-of-care diagnostics, environmental monitoring, food safety, and biomedical research [20, 21].

At the heart of electrochemical biosensors is the electrode interface, or working electrode, where the biorecognition event and subsequent signal generation occur. This electrode, often fabricated from materials such as gold, platinum, carbon, or novel nanostructures like graphene and carbon nanotubes, provides a conductive surface with a high surface area to support the immobilization of the biological recognition element [20]. The bioreceptor is attached to the electrode surface using techniques such as adsorption, covalent bonding, or entrapment within a polymer matrix — the choice of which significantly impacts the biosensor’s stability, sensitivity, and reproducibility. Upon interaction with the analyte, an electrochemical change such as electron transfer, ionic flux, or redox reaction takes place at the electrode, which is transduced into a measurable signal [21]. ECBs can be classified based on the type of signal they generate and they can either be amperometric, potentiometric, voltametric, or impedimetric [22].

####  Amperometric biosensors

Amperometric biosensors measure the current generated at a fixed potential, typically related to a redox process involving the analyte. These biosensors commonly have reaction times, energetic ranges, and sensitivities comparable to the potentiometric biosensors. In amperometric biosensors, the output current of the sensor is analyzed and used for the sensing process [23]. The sensitivity of the amperometric biosensor is determined by comparing the current obtained for the different analyte concentrations.

They are widely used for enzymatic sensing. Owing to its various advantages such as high sensitivity, wide linear dynamic range, fast response time, simple and cost-effective electronics, adaptability to miniaturization, integration and compatibility with signal amplification strategies, has made many researchers to employ amperometric biosensors for various applications, including the detection of glucose [24–26], lactate [27, 28], cholesterol [29–31], *E. coli* [32–34], neurotransmitters-dopamine, serotonin, epinephrine, glutamate, norepinephrine [35–40], Hydrogen peroxide [41, 42], uric acid, Urea [43–45], etc.

For instance, Kausaite-Minkstimiene and research group [46], constructed a reagent-less amperometric glucose biosensor on a graphite rod (GR) modified layer-by-layer with 1,10‑phenanthroline-5,6-dione (PD) and glucose oxidase (GOx). The enzyme catalyzes glucose oxidation, producing H₂O₂, which is electrochemically oxidized at + 0.2 V vs. Ag/AgCl, generating a current proportional to glucose concentration. The amperometric glucose biosensor GR/PD/GOx achieved a detection limit of 0.025 mM, Linear range of 0.1–76 mM, and a high selectivity against ascorbic/uric acids. When analysing human serum samples, the biosensor showed good accuracy, and it was very selective for glucose.

In a similar study conducted by Palanisamy et al., [24], an amperometric glucose biosensor was developed based on glucose oxidase (GOx) immobilized within a multiwalled carbon nanotubes (MWCNTs)/graphene oxide (GO) hybrid biocomposite. The biosensor was fabricated by dispersing GOx in the MWCNTs/GO matrix on a glassy carbon electrode surface. The hybrid matrix provided a high surface area and good electrical conductivity, enhancing electron transfer between the active center of GOx and the electrode. The developed biosensor detected glucose via oxidation of H₂O₂ generated during the GOx-catalyzed oxidation of glucose, operating at a potential of + 0.6 V vs. Ag/AgCl. The glucose biosensor achieved a detection limit of 28 µM and a linear range of 0.05–23.2 mM, demonstrating excellent sensitivity and stability for glucose detection.

Amatatongchai and research group [25], fabricated an amperometric glucose biosensor for flow injection analysis, utilizing glucose oxidase immobilized on a carbon paste electrode (CPE) modified with a nanocomposite of carbon nanotubes (CNTs) and platinum nanoparticles (PtNPs). The use of PtNPs significantly enhanced the electrocatalytic activity toward H₂O₂ oxidation, allowing for effective glucose detection at + 0.45 V vs. Ag/AgCl. This biosensor exhibited a wide linear range of 0.1–3 mM (R^2^ = 0.995) and 5–100 mM (R^2^ = 0.997), with corresponding sensitivities of 0.127 and 0.060 (µA s) mM^− 1^, respectively, and a low detection limit of 15 µM. The flow injection configuration enabled high-throughput and reproducible measurements, and the electrode demonstrated good operational stability and resistance to interference from common species like ascorbic acid and uric acid. The fabricated biosensor was effectively utilized to detect glucose in real samples such as food and pharmaceuticals.

**Fig. 1:**
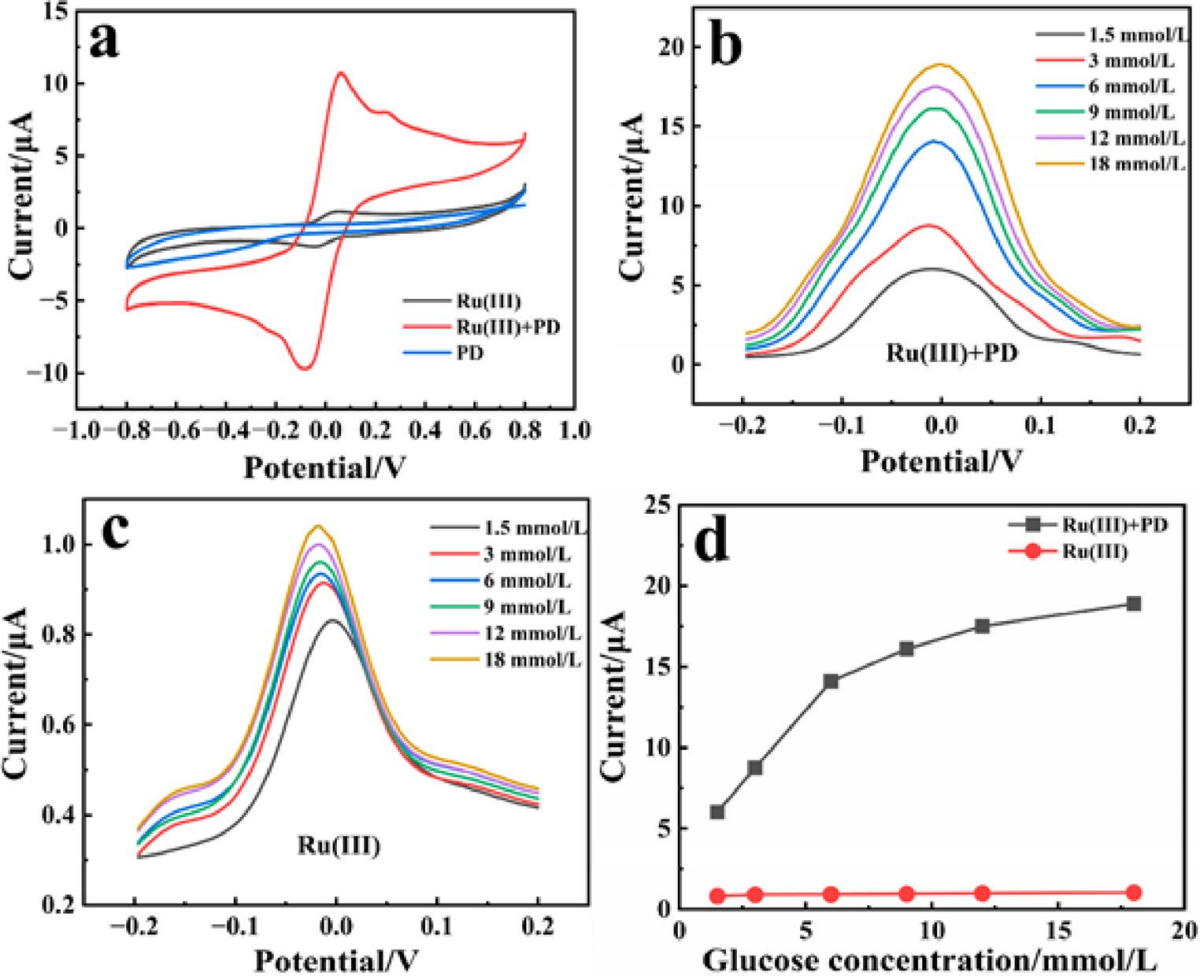
**a** CV of PD, Ru(III), or PD/Ru(III) sensor in 6 mM glucose solution (scan rate = 0.1 V/s),** b** and** c** DPV graph of PD/Ru(III) sensor and Ru(III) sensor, respectively, at various glucose concentrations, and** d** Current response against different glucose concentrations for Ru(III) and PD/Ru(III) sensor [8]

Quan and co-workers [26] reported the design of an amperometric glucose sensor using dual redox mediators (1,10-Phenanthroline-5,6-dione (PD)/Ru(III)) to shuttle electrons between GOx and a modified electrode surface. Figure 1 shows the CV, DPV of the electrode materials. The dual mediators enabled operation at a low working potential of + 0.2 V vs. Ag/AgCl, significantly minimizing interference from electroactive species. The biosensor displayed a detection limit of 7.0 µM and a linear range of 0.01 to 38.6 mM with a sensitivity of 38 µA·L/(mmol·cm^2^). The biosensor demonstrated exceptional high selectivity and stability. The introduction of two mediators provided synergistic effects, leading to faster electron transfer and improved sensitivity compared to single-mediator systems. Equations 1–4 illustrate the glucose redox reaction mechanism catalyzed by the developed sensor PD/Ru(III), where the collaborative impact of PD and Ru(III) enhances the sensor’s response current signal and facilitates electron transfer in the enzyme-catalyzed process.

1 $${\mathrm{Glucose}}\,+\,{\mathrm{FAD}}\, - \,{\mathrm{GDH}} \to {\text{Gluconic Acid}}\,+\,{\mathrm{FAD}}{{\mathrm{H}}_{\mathrm{2}}}\, - \,{\mathrm{GDH}}$$

2 $${\mathrm{FAD}}{{\mathrm{H}}_{\mathrm{2}}}\, - \,{\mathrm{GDH}}\,+\,{\mathrm{PD}} \to {\mathrm{FAD}}\, - \,{\mathrm{GDH}}\,+\,{\mathrm{PD}}{{\mathrm{H}}_{\mathrm{2}}}$$

3 $${\mathrm{PD}}{{\mathrm{H}}_{\mathrm{2}}}\,+\,{\mathrm{2R}}{{\mathrm{u}}^{{\mathrm{3}}+}} \to {\mathrm{PD}}\,+\,{\mathrm{2R}}{{\mathrm{u}}^{{\mathrm{2}}+}}+{\text{ 2}}{{\mathrm{H}}^+}$$

4 $${\mathrm{R}}{{\mathrm{u}}^{{\mathrm{2}}+}} \to {\mathrm{R}}{{\mathrm{u}}^{{\mathrm{3}}+}}+{\text{ }}{{\mathrm{e}}^ - }$$

In another study, an amperometric biosensor was designed by Xu et al., [47] to determine glucose in blood serum. In their research, they self-assembled glucose oxidase and Pt nanoparticles (encapsulated with dendrimers) on a MWCNTs via layer-by-layer (LbL) adsorption approach. The biosensor was very sensitive (30.64 mA mM^− 1^ cm^− 2^) to glucose and had a low limit of detection (LOD) (2.5 mM) with 5 mM – 0.65 mM LDR (Light Dependent Resistor) and swift response time (5 s). The application of amperometric biosensor is also extended to cholesterol detection where Haritha et al., [29] constructed Au/MWCNTs amperometric biosensor for detecting cholesterol. The proposed biosensor recorded a detection limit of 0.5 µM, LDR of 2.0 µM-1.4 mM within 15 s of response time. Other amperometric cholesterol-based biosensors have been reported by numerous researchers with low LOD and good sensitivity [30, 31, 48].

Using a nanocomposite of Ga_2_O_3_-doped ZnO decorated with single-walled carbon nanotubes (Ga_2_O_3_⋅ZnO@SWCNTs), Ahmed et al., [35] reported an amperometric dopamine sensor with high sensitivity, wide linear range, and excellent selectivity. The sensor catalyzes dopamine oxidation at + 0.45 V vs. Ag/AgCl, where dopamine is electrochemically oxidized, generating a current proportional to its concentration. The sensor achieved a LDR of 1.0–2056 µM, sensitivity of 0.2536 µA µM⁻¹ cm⁻², and a detection limit of 0.052 µM. The fabricated biosensor demonstrated rapid response (94% of steady-state current within 4 s), high reproducibility (RSD 3.6% for repeatability), and outstanding selectivity against common interferents like ascorbic acid, uric acid, glucose, and chloride ions. The sensor was effectively applied for dopamine determination in human blood serum and commercial injection samples with recoveries close to 100%.

When comparing with existing dopamine sensors, Ga_2_O_3_⋅ZnO@SWCNT sensor displayed competitive sensitivity and a broader linear range. Notably, the low working potential of + 0.45 V minimized interference, which is a key advantage over many other sensors that operate at higher potentials, reducing selectivity. Overall, the Ga_2_O_3_⋅ZnO@SWCNT/GCE sensor combines the high surface area of SWCNTs, enhanced redox activity from the Ga_2_O_3_⋅ZnO heterojunction, and the benefits of nanocomposite formation for improved imbalanced dopamine (DA) adsorption and electron transfer. Its practical applicability in real sample analysis, stability (retaining 83% initial current after 30 days), and ease of fabrication position it as a promising platform for future electrochemical sensing applications in clinical diagnostics and pharmaceutical analysis. Other neurotransmitters like serotonin, epinephrine, glutamate, norepinephrine have also been detected using amperometric based biosensors [36–38, 49, 50].

Amperometric biosensors offer several advantages that make them highly effective for analytical applications. They are exceptionally sensitive, capable of detecting analytes at nano- to femtomolar levels, and they provide a wide linear dynamic range, allowing for accurate quantification across broad concentration spans. Their fast response times, often within seconds to minutes, result from rapid electron transfer at the electrode surface [51]. Additionally, their operation relies on simple and cost-effective electronics, making them more accessible than spectroscopic methods. Amperometric biosensors are also well-suited for miniaturization, enabling integration into portable and wearable devices for point-of-care testing. Furthermore, their design is compatible with various signal amplification strategies, such as the use of nanomaterials and redox mediators, enhancing their sensitivity even further [52].

Amperometric biosensors, despite their advantages, face several limitations. They are prone to interference from electroactive species in complex samples, which can lead to inaccurate readings as fluctuations can introduce noise or drift. Therefore, maintaining a stable applied potential is crucial [51]. The biological elements, such as enzymes, used in many designs are sensitive to environmental conditions and may degrade over time, affecting performance. Electrode fouling from sample residues or reaction by-products can further impair sensitivity and reproducibility. Additionally, disposable designs often have limited lifespans, raising operational costs. Some sensors also depend on dissolved oxygen levels, which can compromise accuracy in oxygen-variable environments [52].

To address these setbacks, several strategies can be employed. Interference from electroactive species can be minimized using selective membranes, nanomaterial-based filters, or applying differential pulse techniques to distinguish target signals. Maintaining a stable potential can be achieved through advanced potentiostatic circuits and feedback control systems. Enhancing enzyme or bioreceptor stability involves immobilization techniques, such as encapsulation in polymers or use of genetically engineered enzymes with improved resilience. Electrode fouling can be reduced by surface modifications with antifouling coatings or self-cleaning electrode designs. To extend sensor lifespan and reduce costs, reusable electrode platforms and long-lasting biorecognition elements can be used. Finally, oxygen-independent detection methods or the use of artificial electron acceptors can mitigate oxygen dependency issues in relevant applications.

#### Potentiometric biosensors

Potentiometric biosensors measure the change in potential at zero current, typically relying on ion-selective electrodes that sense ionic activity changes. This technique is advantageous in that it is simple, compact, and requires little power. Additionally, the negligible current flow means the technique should be more resistant to interferent effects and ohmic drop considerations compared to voltametric or amperometry sensors. Potentiometry has shown to be relatively insensitive to electrode size, meaning that miniaturization without loss of sensitivity is achievable [53]. These have been used for detection of numerous molecules such as ascorbic acid, uric acid, cysteine [54], serotonin [55], valproic acid [56], Alzheimer biomarker [57], glucose [58], urea [59], galactose [60], and many others. They are also useful in detecting ions like Na^+^, K^+^, and Ca^2+^ or hydrogen (pH sensors) [61].

For instance, Freeman and research group [54] fabricated miniaturized nanoporous gold (NPG) electrodes for potentiometric biosensing of ascorbic acid (AA), uric acid (UA), and cysteine (Cys) in microliter volumes. The electrodes, prepared by dealloying 12 K gold leaf and attaching it to glass capillaries, exhibited high surface area (~ 16× the geometric area) and enabled open-circuit potential (OCP) measurements in 100 µL solutions. The biosensor demonstrated Nernstian-like behaviour with sensitivities of ~ − 31 mV/decade for AA, − 26.5 mV/decade for Cys, and − 29 mV/decade for UA across concentration ranges of 50–2000 µM (AA), 100–1500 µM (Cys), and 100–2000 µM (UA). In mixed solutions mimicking physiological conditions, the OCP was predominantly influenced by AA concentration, highlighting the sensor’s potential for redox monitoring in complex biological samples. The fabricated biosensor was effectively utilized to assess redox potential in small-volume biological mimics relevant to blood and plasma analyses.

In a related study, Öndes et al., [59] reported the design of a high-stability potentiometric urea biosensor based on poly(HEMA-GMA) nanoparticles covalently attached with urease enzyme. The sensor detects urea by enzymatic hydrolysis into ammonium ions, which are measured potentiometrically using an ammonium-selective electrode. The biosensor exhibited a wide LDR of 0.01–500 mM and a low LOD of 0.77 µM, with a rapid response time of 30 s. The system demonstrated high repeatability, retaining 95.7% activity after 170 cycles, and excellent storage stability with 83% activity after 90 days. Importantly, the biosensor showed negligible interference from common substances such as ascorbic acid, uric acid, glucose, and sodium ions, and was successfully applied for urea determination in artificial serum samples.

Potentiometric biosensors offer several advantages that make them highly suitable for diverse applications. They consume very low power because they operate at near-zero current, making them ideal for portable and wearable devices. Their instrumentation is simple and cost-effective, as they do not require complex electronics for signal detection [62]. These biosensors are also highly compatible with miniaturization technologies, such as ISFETs.

ion-sensitive field-effect transistor) and screen-printed electrodes, allowing integration into lab-on-a-chip and point-of-care systems. They support continuous, real-time monitoring with minimal need for recalibration and can be tailored to detect a wide range of analytes using selective membranes or enzymatic layers. Additionally, their non-destructive measurement approach preserves the integrity of sensitive biological or environmental samples [63].

Potentiometric biosensors, while advantageous in many respects, also face notable limitations. They generally exhibit lower sensitivity and higher detection limits compared to amperometry sensors, making them less suitable for trace analyte detection. Their readings can be affected by electrical noise, temperature fluctuations, and potential drift, requiring frequent recalibration. Without highly selective membranes or recognition layers, these sensors are prone to interference from similar ions, reducing specificity. Their response times are typically slower, particularly when reliant on ion diffusion through membranes. Additionally, variations in the sample matrix—such as pH, ionic strength, or the presence of proteins—can impact measurement accuracy. Finally, the ion-selective membranes used in these sensors are often fragile and susceptible to degradation or fouling, which can limit their operational lifespan [63].

To address the demerits of potentiometric biosensors, several strategies can be implemented. Enhancing sensitivity can be achieved by incorporating nanomaterials or optimizing membrane compositions to improve signal transduction. Drift and noise issues can be minimized through temperature compensation, shielding from electromagnetic interference, and the use of stable reference electrodes. Improving selectivity involves developing high-quality ion-selective membranes or incorporating highly specific biorecognition elements like enzymes or aptamers. Response times can be shortened by using thinner or more porous membranes to accelerate ion diffusion. Matrix effects may be mitigated through sample pretreatment, buffering, or membrane coatings that resist pH and ionic fluctuations. Lastly, membrane longevity can be extended by using more robust, chemically resistant materials and antifouling surface treatments to maintain consistent performance over time.

#### Voltametric biosensors

Voltametric biosensors are electrochemical devices that detect target analytes by measuring the current response resulting from redox reactions as a function of an applied potential. These biosensors typically use enzymes, antibodies, nucleic acids, or other biorecognition elements immobilized on an electrode surface to catalyse or facilitate specific interactions with the analyte. The resulting electron transfer generates a current signal proportional to the analyte concentration, measured using techniques like cyclic voltammetry, differential pulse voltammetry, or square-wave voltammetry [64]. Voltametric biosensors offer high sensitivity, the ability to probe redox properties of analytes, and flexibility in detecting a wide range of biological and chemical species [65]. They are widely used in clinical diagnostics, food safety, and environmental monitoring. However, challenges such as potential interference from electroactive species in complex matrices, electrode fouling, and the need for careful control of experimental conditions can affect performance and require optimization for reliable operation [66]. These biosensors have been used for detection of numerous molecules such as paracetamol [67], cardiac troponin biomarkers [68], SARS-CoV-2 Proteins [69], serotonin [70], miRNAs [71], etc.

Goyal and co-workers [67] developed a voltametric biosensor by modifying an edge plane pyrolytic graphite electrode (EPPGE) with single-walled carbon nanotubes (SWCNTs), enabling highly sensitive paracetamol detection. Using square-wave voltammetry in a pH 7.2 phosphate buffer, the sensor achieved a well-defined oxidation peak at ≈ + 187 mV (vs. Ag/AgCl) and demonstrated a remarkable LDR from 5 to 1,000 nM, with an ultra‑low limit of detection of 2.9 nM. The single-walled carbon nanotubes (SWCNTs) enhancement led to doubled sensitivity compared to multi-walled carbon nanotubes (MWCNTs) and facilitated rapid, adsorption-controlled, two-electron reversible oxidation. Selectivity tests confirmed minimal interference from common physiological compounds, and the method was validated with paracetamol tablets and human urine samples, affirming its practicality for real-world paracetamol monitoring.

In another study, Goya and team [69] reported a rapid, ultrasensitive voltametric biosensor for detecting severe acute respiratory syndrome coronavirus 2 (SARS-CoV-2) spike protein using a functionalized graphene oxide–modified glassy carbon electrode (BSA/antibody/f-GO/GCE or SPE). The sensor performed antigen–antibody recognition, generating distinct oxidation signals at approximately − 200 mV on screen-printed carbon electrode (SPE) and + 1430 mV on glassy carbon electrode (GCE). It demonstrated an exceptionally low detection limit of 1 attogram per milliliter (ag/mL) and a dynamic linear range from 1 ag/mL to 10 fg/mL, with detection achieved in just 5 min screen-printed carbon electrode (SPE) and 35 min (GCE). When tested with saliva and oropharyngeal swab samples, the sensor achieved 93.3% sensitivity and 92.5% specificity compared to reverse transcription and polymerase chain reaction (RT-PCR), with the SPE version achieving 91.7% accuracy, outperforming a rapid antigen test kit (66.7%). The exceptional sensitivity and rapid response of this voltametric biosensor underscore its potential for real-world COVID-19 diagnostics in complex biological matrices.

Voltametric biosensors offer a range of advantages that make them highly effective for analytical applications. They exhibit high sensitivity, enabling detection of trace and ultra-trace levels of analytes, and support a wide dynamic range for quantifying substances across broad concentration spans [72]. These sensors provide rich electrochemical information, including redox potentials and electron transfer kinetics, which enhances analytical insight. They are versatile, suitable for detecting a variety of redox-active compounds such as drugs and metabolites. Advanced techniques like differential pulse and square-wave voltammetry enhance resolution and signal-to-noise ratio. Voltametric biosensors also offer rapid response times, are easily enhanced with nanomaterials like carbon nanotubes or graphene, and support multi-analyte detection by adjusting scanning parameters or potential windows [72].

Voltametric biosensors, despite their strengths, come with several drawbacks. They are prone to interference from electroactive species in complex samples, which can distort or obscure target signals. Electrode fouling due to adsorption of by-products or biomolecules can diminish sensor performance over time. Their operation often requires skilled handling, including careful control of potential, scan parameters, and interpretation of data. Calibration can be complex and needs to be done frequently to maintain accuracy. Additionally, the instrumentation is generally more expensive and sophisticated than that required for simpler methods like potentiometry. Long-term stability may also be an issue, as sensor performance can degrade without regular maintenance and care [22].

To overcome the limitations of voltametric biosensors, several strategies can be employed. Interference from electroactive species can be minimized through selective membranes, advanced signal processing, or the use of modified electrodes that enhance target specificity. Electrode fouling can be reduced by surface modifications with antifouling coatings or by employing disposable electrode strips. Operator dependency and complexity can be addressed by integrating automated systems with user-friendly interfaces and pre-set scan protocols. Frequent calibration requirements may be mitigated using internal standards or self-calibrating systems. While instrumentation can be costly, advancements in microelectronics and portable electrochemical analyzers are making voltametric systems more accessible and affordable. Lastly, long-term stability can be improved by using robust electrode materials (e.g., gold, carbon nanostructures), protective coatings, and implementing regular maintenance protocols to ensure consistent performance over time.

#### Impedimetric biosensors

Impedimetric biosensors are electrochemical devices that detect biological analytes by measuring changes in the electrical impedance of a system, typically at the interface between an electrode and a solution [73]. These biosensors operate by monitoring how the binding of a target molecule (such as an enzyme substrate, antibody-antigen pair, or DNA hybridization product) alters the impedance characteristics—specifically resistance and capacitance—at the electrode surface, often over a range of frequencies using electrochemical impedance spectroscopy (EIS). They offer several advantages, including label-free detection, high sensitivity, and real-time monitoring of binding events. Impedimetric biosensors have gained attention for label-free detection in immunosensors and DNA sensors due to their ability to monitor binding events without the need for redox-active labels [73, 74]. They are widely used in clinical diagnostics, environmental monitoring, and food safety due to their ability to detect low concentrations of analytes without requiring complex sample preparation. However, they can be susceptible to signal drift and interference from non-specific adsorption and require precise control of experimental conditions and electrode surface properties for reliable performance. Numerous research studies have been reported on the usage of this biosensor for determination of enrofloxacin [75], bromate [76, 77], bisphenol A [78], mycotoxin zearalenone [79], melatonin [80], ochratoxin A [81], HIV-1 DNA [82], tetracycline [83], kanamycin [84], and lot more.

**Fig. 2:**
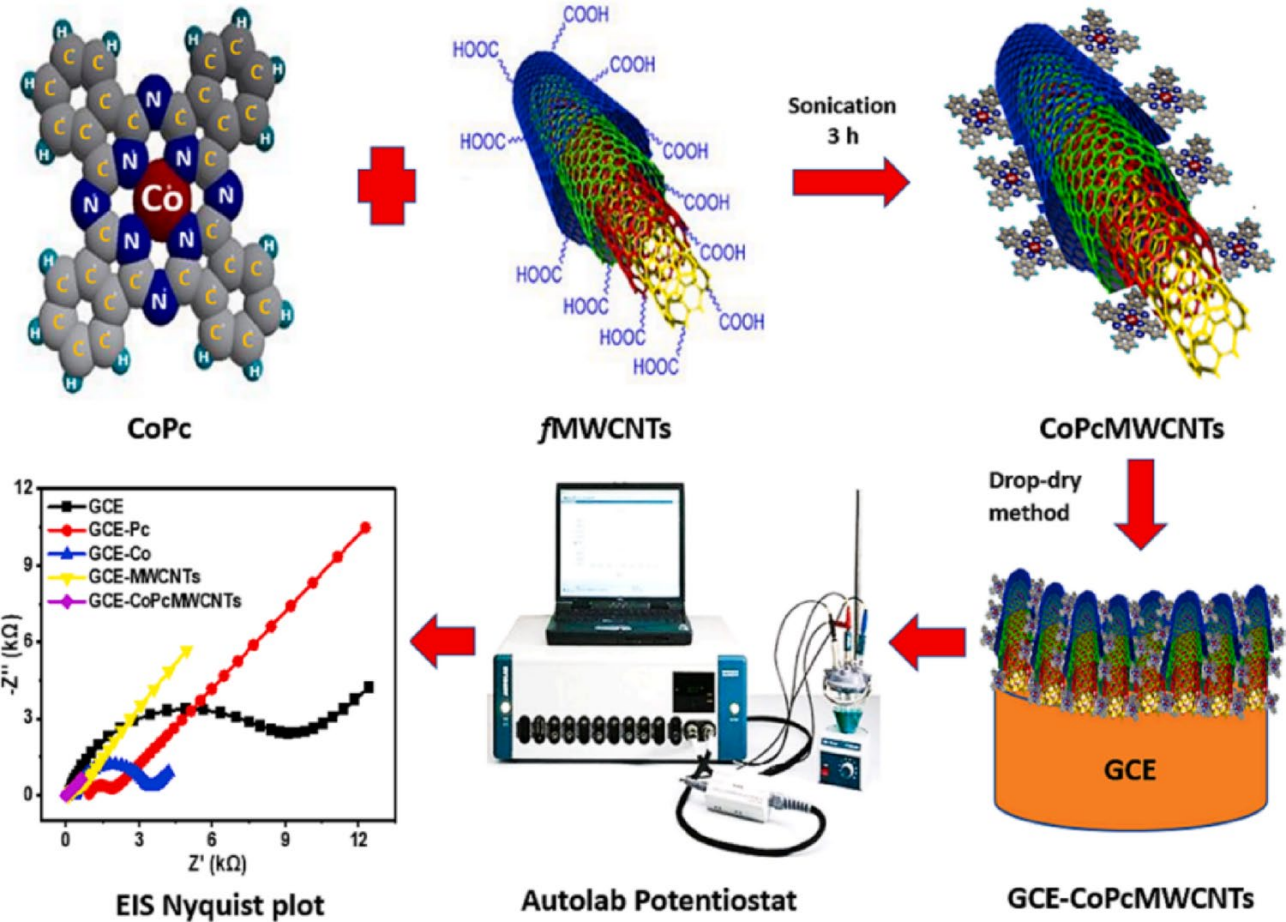
Synthesis and fabrication process of GCE-CoPcMWCNTs biosensor. Reprinted with permission from [85].

For example, Balogun and Fayemi [76] developed an impedimetric and voltametric sensor for bromate detection by modifying a glassy carbon electrode (GCE) with a cobalt phthalocyanine–multiwalled carbon nanotube (CoPc-MWCNTs) nanocomposite (Fig. 2). Electrochemical impedance spectroscopy (EIS) and square-wave voltammetry (SWV) were employed in 0.1 M H₂SO₄ (pH 1), with EIS achieving a low LOD of 0.32 µM and a sensitivity of 72.4 µM⁻¹ kΩ⁻¹ over a linear range of 1–8 µM, while SWV delivered an LOD of 1.31 µM and a high sensitivity of 497.6 µA µM⁻¹ across 6–20 µM (LDR). The sensor exhibited excellent analytical performance, showing good selectivity, strong stability (retaining 93.5% of its initial signal), and reproducibility with a relative standard deviation of 3.9%. Practical applicability was demonstrated through successful detection of bromate in real bread samples with satisfactory recovery rates. The enhanced performance is attributed to synergistic effect between the CoPc and the fMWCNTs, which improves electron transfer rates and surface area.

In a related study, the same authors–Balogun and Fayemi [77] also fabricated GCE-NiPc-MWCNTs for impedimetric and voltammetric bromate sensor. In their findings, the developed multi-function biosensors achieved LOD of 6.72 µM with a sensitivity of 483.7 µA µM⁻¹ across a wide linear range of 24–100 µM when utilizing EIS in 0.1 M H₂SO₄ (pH 1) and provided a lower LOD of 1.47 µM, a high sensitivity of 1293 µA µM⁻¹, and a LDR of 12–56 µM. The method used to fabricate this sensor is shown in Fig. 2.

Impedimetric biosensors offer several notable advantages, making them versatile tools in biosensing. They enable label-free detection of biomolecular interactions, eliminating the need for complex labelling steps and reducing costs. Their high sensitivity to surface changes allows for the precise monitoring of events like antigen–antibody binding or cell layer formation. These sensors support non-destructive, real-time measurements, preserving sample integrity while allowing continuous monitoring [86]. They are applicable to a wide range of targets.

One of the advantages of impedimetric biosensing is that it can be used for sensing small molecules, entire cells and pathogens, depending on the biorecognition layer used. Impedimetric biosensors are also highly compatible with miniaturization and can be easily integrated into microfluidic chips and portable devices. Additionally, their flexible transducer designs—such as interdigitated or nanostructured electrodes—enhance sensitivity and allow customization for specific analytical needs [87].

Impedimetric biosensors face several limitations that can impact their performance. They are susceptible to non-specific binding and electrode fouling, which can lead to false positives and reduced accuracy. Variations in sample conditions like pH, ionic strength, or viscosity can influence impedance readings, complicating data interpretation. Analysing impedance spectra often requires complex modelling and can be mathematically demanding. These sensors are also sensitive to temperature fluctuations, necessitating strict thermal control for reliable results. Additionally, they may require long equilibration times for certain analytes and involve costlier, more complex instrumentation compared to simpler electrochemical methods.

To address the demerits of impedimetric biosensors, surface modification with antifouling coatings or selective membranes can reduce non-specific binding. Buffering and calibration strategies help mitigate sample matrix effects, while temperature control or compensation improves measurement stability. Advanced software and automated fitting tools simplify impedance data analysis, and miniaturized, cost-effective analyzers are making the technology more accessible. Examples of the electrochemical biosensors with their applications are shown in Table 2.

**Table 2:** Summary of electrochemical biosensors and their applications

Electrode	Analyte	Sample	Technique	LOD (µM)	LDR (µM)	References
GR/PD/Gox	Glucose	Human serum	Amperometric	0.025 mM	0.1–76 mM	[46]
MWCNT/GO/Gox	Glucose	glucose oxidase	Amperometric	28 µM	0.05–23.2 mM	[88]
CNTs–PDDA–PtNPs/CPE	Glucose	food & pharmaceuticals	Amperometric	15 µM	0.1–3 mM 5– 100 mM	[25]
PD/Ru(III)	Glucose	Human blood	Amperometric	7.0 µM	0.01–38.6 mM	[26]
Pt/MWCNTs/Gox	Glucose	Human serum	Amperometric	2.5 mM	5–0.65 mM	[47]
Au/MWCNTs-GCE	Cholesterol	-	Amperometric	0.5 µM	2 µM − 1.4 mM	[29]
Ga₂O₃⋅ZnO@SWCNT	Dopamine	Blood serum	Amperometric	0.052 µM	1.0–2056 µM	[35]
NPG	Ascorbic Acid Uric Acid Cysteinen	-	Potentiometric		50–2000 Μm 100–2000 µM 100–1500 µM	[54]
Poly(HEMA-GMA) NP	Urea	Human serum	Potentiometric	0.77 µM	0.01–500 mM	[59]
EPPGE/MWCNTs	Paracetamol	Human urine	Voltammetric	2.9 nM	5-1000 nM	[67]
BSA/AB/f-GO/GCE	COVID-19	SARS-CoV-2 spike protein	Voltammetric	1 ag/mL	1 ag/mL-10 fg/mL	[69]
CoPc-MWCNTs/GCE	Bromate	Bread Bread	Impedimetric Voltammetric	0.32 µM 1.31 µM	1–8 µM 6–20 µM	[76] [76]
NiPc-MWCNTs/GCE	Bromate	Bread Bread	Impedimetric Voltammetric	6.72 µM 1.47 µM	24–100 µM 12–56 µM	[77] [77]

A significant advancement in electrochemical biosensors is the integration of nanomaterials to enhance performance. Nanostructures like carbon nanotubes, graphene, metal nanoparticles, and metal oxides can dramatically increase the electrode’s surface area, improve electron transfer kinetics, and facilitate the immobilization of bioreceptors. This leads to improved sensitivity, lower detection limits, and faster response times. Additionally, nanomaterial-functionalized electrodes can exhibit unique catalytic or adsorptive properties that further enhance biosensor performance.

Another strength of electrochemical biosensors is their compatibility with microfabrication and lab-on-a-chip technologies. Electrochemical detection systems can be readily miniaturized and integrated into portable, disposable devices for on-site and real-time analysis. This has been particularly transformative for medical diagnostics (e.g., portable glucose meters, wearable sensors), environmental monitoring (e.g., detection of pollutants in water), and food quality assessment (e.g., detection of pathogens or toxins). Moreover, the low power requirement and potential for wireless data transmission make electrochemical biosensors suitable for remote sensing and continuous monitoring applications [89].

Despite these advantages, electrochemical biosensors face challenges related to stability, selectivity, and fouling of the electrode surface. Biological recognition elements can degrade over time, and non-specific binding of interfering species can obscure measurements, especially in complex samples like blood or wastewater. Strategies to overcome these issues include the use of synthetic recognition elements (e.g. molecularly imprinted polymers, aptamers), surface passivation techniques, and incorporation of advanced signal processing algorithms to distinguish specific signals from background noise.

The future of electrochemical biosensors is promising, with ongoing research focusing on multi-analyte detection, integration with flexible and wearable electronics, and coupling with artificial intelligence for intelligent diagnostics [90]. Furthermore, developments in bioelectronics and synthetic biology could enable the design of hybrid biosensors capable of not only detecting but also responding to analytes in a controlled manner (e.g., smart drug delivery systems). Overall, electrochemical biosensors represent a critical bridge between biology and electronics, offering versatile platforms for the next generation of biosensing technologies.

### Mass-based biosensors

Mass-based biosensors are kinds of biosensors that depend on a piezoelectric crystal or cantilever beam for identifying changes in the sensors’ mass surface when a biological substance (such as antibody or aptamer) attaches to them. The presence of target analytes in the biological sample is then indicated by a signal that is created by detecting and converting the change in frequency of the piezoelectric crystal or cantilever beam. Since mass-based biosensors work on the basis of the sensors’ piezoelectric characteristics, binding actions and the corresponding mass increase at the sensor surface may be measured by altering the surface acoustic wave’s oscillation [91]. Among the aforementioned types, piezoelectric quartz sensors are the most extensively used mass sensors owing to their short detection times, affordability, accessibility, and ability to produce label-free sensors [92]. Its mechanical structure involves the attachment of cantilever biosensors to one end of the biosensor. Using common micromachining methods, these sensors may be mass-produced in batches. They provide better reaction, much smaller size, high accuracy, and more dependability than traditional sensors [92].

#### Mass spectrometry biosensing (MS)

Mass spectrometry (MS) is an advanced mass-based analytical method capable of identifying multiple substances at the femtomolar level, exhibiting a wide dynamic range [93–95]. Owing to the capabilities of label-free and high-throughput assays, diverse analytes may be identified using various mass spectrometry methods with exceptional mass resolution [96]. However, the effectiveness of this mass spectrometric biosensing is very restricted when evaluating high-molecular-weight compounds with low ionization rates [97, 98]. Therefore, there is need for more research on signal amplification of MS-based for enhanced detection sensitivity. Also, there is need for validation of target analytes across diverse experimental protocols to increase detection selectivity.

## Application of gold/carbon-based materials in biosensing

The composite form from gold and various carbon-based materials has been widely explored for sensing various biological samples. In this section, the use of the gold/carbon-based materials in detecting oligonucleotides/DNA, tumour cells, protein/enzymes, glucose, thiol, peroxide and microbes such as bacteria and viruses is discussed in detail.

### Sensing of DNA and oligonucleotides using gold/carbon-based materials

Clinical potential and significance of deoxyribonucleic acid (DNA) and oligonucleotides biosensors cannot be overemphasized. They are currently drawing attention in the field of medicine, food analysis, and environmental monitoring due to their versatility, sensitivity and accuracy [99]. They can either be electrochemical electrodes, aptamers, or fluorescent sensors. DNA biosensors can operate by measuring the signals produced during the hybridization process of DNA. After which hybridization of the sample probe to the target DNA sequence is identified through a carefully selected, suitable hybridization indicator [100]. *Helicobacter pylori* (*H. pylori*), a prevalent bacterial infection in humans which can also be responsible for upper gastro duodenal diseases, was detected via a sensitive electrochemical DNA biosensor designed from AuNPs and graphene oxide (GO), using glassy carbon electrode as support. Biosensor takes the advantage of the synergy between the outstanding electric conductivity of GO/AuNPs and the electroactivity of Oracet blue (OB). The detection limit for *H.pylori* was found to be 27.0 mM while the linear range of 60.0-600.0 pM was recorded [100].

**Fig. 3:**
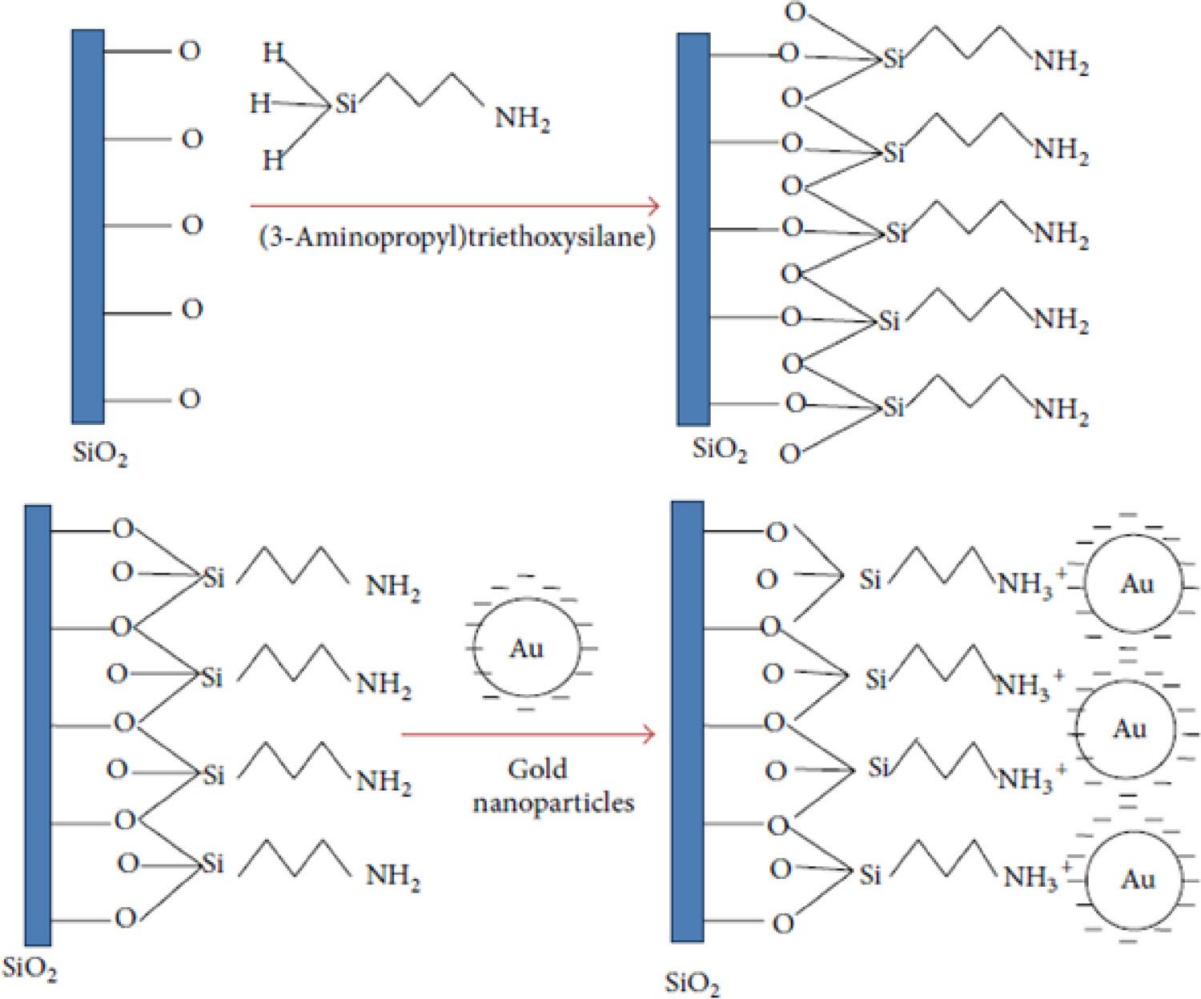
Hypothetical equations for surface of treatment of SiO_2_ by gold nanoparticles and (3-aminopropyl)triethoxysilane (APTES) [101]

The design of an electrochemical genosensor for DNA was also reported by Mohammed et al., [101]. The genosensor was fabricated using AuNPs and (3-aminopropyl) triethoxysilane (APTES) while the thin films of thermally oxidised SiO_2_ served as the support. Modification of the SiO_2_ with a mixture of APTES and AuNPs for the detection of DNA was carried out at 30 min, 1 h, 2 h, and 4 h, respectively. The best result for DNA immobilization and hybridization as revealed in the value of capacitance and permittivity measurement (44 µF and 1450 × 10^3^, respectively) were obtained at time interval 30 min. Figure 3 shows the modification of SiO_2_ with AuNPs and 3-aminopropyl) triethoxysilane (APTES).

**Fig. 4:**
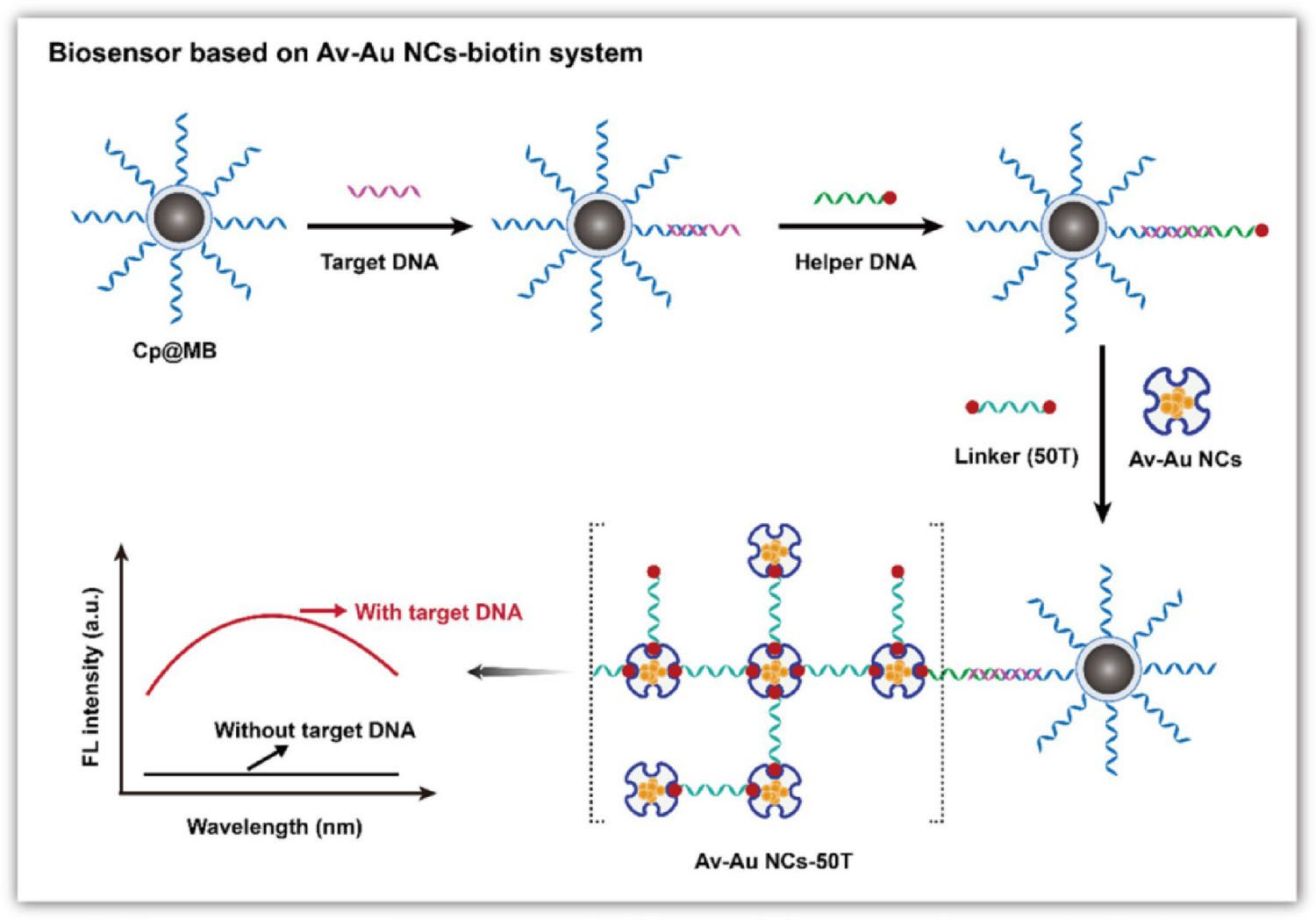
Illustration of the mechanism for detection of the target deoxyribonucleic acid (DNA) by the fluorescence biosensor [103]

In another related study, citrate-capped AuNPs were prepared and used for the fabrication of highly sensitive and versatile DNA biosensor. This biosensor contains self-assembled monolayer of ssDNA and DNA-directed immobilization of DNA-protein conjugates. The macromolecule-decorated AuNPs was connected to a miniaturized gel electrophoresis chip integrated combined with online thermal lens spectrometry (MGEC-TLS) which enabled it to recognise the human epidermal growth factor receptor 2 consisting of extracellular domain (Her2-ECD), an antigen associated with cancer [102]. The biosensor was reported to be very sensitive as a prospective breast cancer biomarker to the extent of detecting HER2-ECD to the concentration of 440 ng/mL. Tang et al., (2022) [103] also reported the performance of an enzyme-free fluorescent biosensor using avidin-stabilized gold nanoclusters (Av-Au NCs). The working principle of the biosensor centres on the amplification of the fluorescent signal emanating from the interaction of Av-Au NCs with biotin. The role played by biotin in this system includes formation of strong covalent bond with gold nanoclusters thereby ‘docking’ it towards the target DNA, which was detected in-vitro. The fabricated fluorescent biosensor was able to detect the target DNA as far as molar concentration of 0.043 nM with the detection limit ranging from 0.2 nM to 20 µM [103]. The process of detection of the target DNA by the fluorescent biosensor fabricated from Av-Au NCs-biotin is illustrated in Fig. 4.

**Fig. 5:**
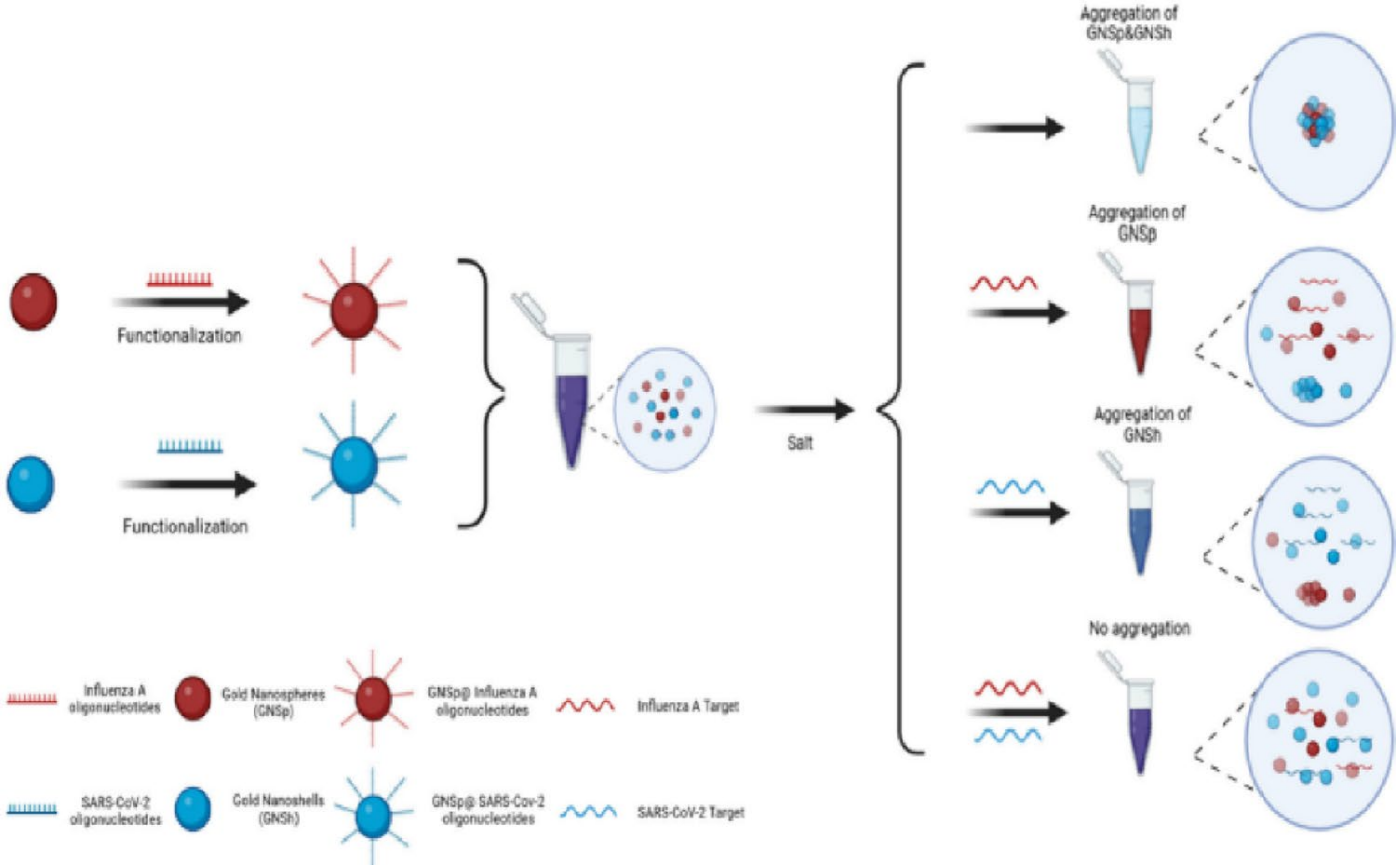
Diagrammatic illustration of process involve in multiple analyte detection using various forms of gold nanoparticles [104]

The performance of a hybrid system of biosensor was examined by Zhang et al., [104]. The hybrid system (Multiplex) containing polymorphous forms of functionalized AuNPs is capable of simultaneously detecting influenza A virus and SARS-CoV-2 virus with analytical sensitivity up to 100 nM, within short time. This is indeed a breakthrough in the field of biosensing because most the studies reported earlier only centred on single target DNA detection. The two gold polymorphs, gold nanospheres and gold nanoshells, were respectively functionalized with the oligonucleotides of the viral SARS-CoV-2 and Influenza A. The limits of detection of 10 and 33 nM were observed in SARS-CoV-2 and Influenza A, respectively. The setup was fascinating to the extent of simultaneously detecting two diseases without loss in specificity and precision, within 20 min of investigation [104]. Figure 5 shows the demonstration of the scheme involved in the detection of multiple analytes using different forms of gold nanoparticle.

### Tumor cell detection using gold/carbon-based materials

The primary benefits of AuNPs as sensors are their biological compatibility and the possibility that their surface will be properly functionalized with a variety of ligands for the selective attachment of molecules and ions [105]. Tang and co-workers designed a novel nanomaterial for the detection of circulating tumor cells (CTCs) using Pt@Ag nanoflowers (pt@AgNFs) and AuNPs/Acetylene black (AuNPs/AB). The created material was used as a substrate to raise the gold electrode’s specific surface area and conductivity. According to the authors, the sensor’s detection ranged from 20 to 1.0 × 10^6^ cells/mL, with limit of detection of 3 cells/mL [106]. Additionally, Darestani-Farahani and colleagues designed a DNA nano biosensor with a sensitive fluorescence characteristic that can identify the DNA sequence of the well-known tumor suppressor gene APC (adenomatous polyposis coli). The authors coupled a fluorophore to AuNPs to boost the nano biosensor’s sensitivity. With an excitation wavelength of 360 nm, the manufactured DNA nano biosensor showed a fluorescence emission at 477 nm. The APC gene’s detection limit was determined to be 1.3 × 10^− 11^ mol/L [107].

Furthermore, biomarkers that detect somatic mutations are also utilized to diagnose possible genetic illnesses as a prophylactic approach. For example, the BRCA1 mutation is one of breast and ovarian cancer which are the frequently occurring oncogenes [108]. It was observed that a DNA capture probe coated in AuNP may be employed to detect these new prognostic biomarkers, with detection approaching femtomolar levels [109]. A developed Pt@Au-nanoring@DNA nanocomposite was also discovered to be beneficial as a diagnostic technique owing to its photothermal treatment properties for tumor cells when subjected to near-infrared radiation [110]. In addition, Bai et al., reported that employing Bi_2_Se_3_-AuNPs as a load signal probe to develop a sandwich with BRCA1-immobilized silicon increases diagnostic accuracy [111].

A FRET immunosensor was developed by Mohammadi and colleagues to identify the CA 15 − 3 tumor marker in human blood and breast cancer cells. AuNPs labelled PAMAM-Dendrimer/aptamer and carbon dots functionalized with antibodies were used in designing the biosensing material. Authors reported that healthy human serum sample was spiked with two concentrations of CA 15 − 3 and the recovery values ranged from 96.97 to 95.86%. In addition, the detection of MDA-MB-231 cell lines in breast cancer was applied to illustrate the usefulness of the proposed material in biomedical area. Results demonstrated linear correlation between the number of MDA-MB-231 cell levels, ranging from 1.0 × 10^3^ to 4.0 × 10^4^ cells/mL (*R* = 0.9955) and LOD of 300 cells/mL [112]. These findings indicated a robustness of data and specificity in the identification of biomarker and cancer cells. FRET immunosensor should be explored for the detection of numerous biomarkers and tumor cells in biological samples. Based on findings, few studies have documented the detection of tumor cells as compared to other applications. Therefore, there is need for further studies on the detection of tumor cells and biomarkers in various biomolecules using functionalized AuNPs biosensors.

### Protein/enzymes detection using gold/carbon-based materials

Functionalized AuNPs have remarkable stability, little resistance, an elevated surface-to-volume ratio [105], and exceptional conductivity [113]. These characteristics make them an optimal material for linking biorecognition systems with signal transduction, and serve as a vital component in the design and execution of biological detection devices [114]. Ercole et al., [115] conducted research on early stress detection in forest trees, whereby gold nanoparticles (AuNPs) were electrochemically coated by drop-casting tetrachloroauric acid solution (1 mM) in H_2_SO_4_ (0.5 M) and applying 0.18 V for 50 s. This method was used to identify the presence of ascorbate peroxidase (APX) in protein extracts from both *P. sativum* and *P. nigra*. Authors documented the effective measurement of APX activity elicited by stress conditions in the foliage of plants cultivated in soil-polluted with heavy metals [115]. This study is a crucial one in the field of environmental pollutant/stimuli identification and control. However, there is need for more studies on other relevant biomarkers that could be employed to evaluate stress conditions in plants. The improvement will facilitate the identification of antibodies capable of cross-reacting with plant antigens and assist in monitoring their physiological conditions and mitigating decline.

Nazarpour et al., [116] used reduced graphene oxide/gold nanoparticles (AuNPs/rGO) to create a unique electrochemical sensor. The nanocomposite was used to detect tryptophan, which serves as an effective biomarker for lung cancer. The concentration of tryptophan detected ranged was from 0.5 to 500 µmol/L, while the LOD was 0.39 µmol/L [116]. Authors concluded that the developed electrochemical sensor is appropriate for quantifying tryptophan in human plasma, serum, and saliva. Di Natale and co-workers have reported picogram-level detection of Tau protein intermediate aggregates employing AuNP-functionalized polymer film as sensing component. The LOD of the protein was 460 pg/mL, while the limit of quantification (LOQ) of 1.1 ng/mL was achieved. An intriguing aspect of the study is that, authors adopted a method which was based on a colorimetric test without utilizing expensive equipment that requires skilled workers when compared to studies from the literature [117]. Authors concluded that the biosensor might be employed in small and portable devices, because it requires low volume of biological sample to be recognized. This research indicate that the Tau protein was totally aggregated at basic pH, hence there is a gap to be solved when the biosensor is employed in acidic pH. This will assist in detecting aggregates that exist in either acidic or basic medium of biological fluids such as saliva and urine. Similar work has been reported by Olorundare et al., [118] using gold nanoparticles with carbon nanofiber for the detection of the alpha - fetoprotein cancer biomarker.

Furthermore, Ferreira et al., [119] created a new biosensor for detecting creatine kinase (CK-MB) in biological samples using gold nanoparticles stabilized with cysteamine, and functionalized with creatine phosphate. The Surface Plasmon Surface (SPR) technique used for analysis revealed that the LOD, LOQ, and sensitivity for CK-MB in the biological samples were 0.209 ng/mL, 0.696 ng/mL, and 8.67 × 10^–3^ ± (◦) mL/ng, respectively [119]. Apart from rapid time of analysis achieved by the biosensor, authors also reported that the biosensor was developed using small amount of materials, thereby reducing its cost of production. This study confirms that functional and efficient biosensors could be designed utilizing low-cost materials. The gap of optimization using various amino acids with higher stability is yet to be filled in this research; thus, there is need for future research. Zhao et al., [120] created a self-powered biosensor (ITO-PAN-CNT–COOH–AuNPs substrate electrode) from polyacrylonitrile-carbon nanotube (PAN-CNT), indium tin oxide (ITO) electrode, and AuNPs. The designed biosensor was employed for the detection of myoglobin. Results showed that the sensor’s range of linearity varied from 5 to 5.0 × 10^3^ ng/mL, while the LOD of 0.23 ng/mL was achieved [120]. Authors reported that the designed biosensor demonstrated excellent stability and selectivity. Therefore, this sensing material could be used without external power supply and finds application in medical application. The concentration of myoglobin in blood sample could be determined and subsequently used for muscle diagnosis. Based on findings, there is need for developing new biosensors by employing recent biomaterial that would improve sensitivity in either acidic or basic medium at reduced time.

### **Heavy metal ions detection using gold/carbon-based materials**

Majority of the metal ions causes serious health problems, therefore their detection in different media is necessary. Composite of gold/carbon materials has also played a role in detecting the heavy metals ions [121]. In a study, gold-carbon composite electrode was synthesized by using gold particles, carbon-epoxy electrodes, carbon paste, and plane graphite. This was then used to detect the presence of trivalent arsenic. With the presence of micron-sized gold array particles in the electrode, high arsenic sensitivity of 10(± 0.1) A mol^− 1^ L and LOD of 5(± 2)×10^− 9^ mol L^− 1^ were obtained [122]. Another heavy metal ions that have been detected using gold/carbon composite is divalent mercury. This was carried out through stripping voltammetry by incorporating gold nanoparticles into graphene oxide functionalized with ionic liquid. This material showed a good linear range of 0.1–100 nM with limit of detection of 0.03 nM. Interestingly, there was no significant interference effect when the detection of divalent mercury was carried out in the presence of other ions that co-existed in the test solution [123]. Another carbon material that has been incorporated into gold nanoparticles for sensing divalent mercury is carbon nanotube. Just like the study carried out with gold/graphene oxide functionalized with ionic liquid, mercury was detected via anodic stripping voltammetry. This composite modified on glassy carbon electrode gave a limit of detection of 3 × 10^− 10^ mol/L and wide linear range of (5 × 10^− 10^–1.25 × 10^− 6^ mol/L) [124]. This has shown that the limit of detection of mercury can be enhanced by changing the carbon-based compounds incorporated into gold for its detection. Apart from mercury, hexavalent chromium has also been detected using gold/carbon-based materials. In a study, nanocomposites of gold and chitosan was used to detect hexavalent chromium in aqueous system, sensitivity and limit of detection of 0.022 nm/ppm and 10 ppm were observed respectively [125].

### Glucose detection using gold/carbon-based materials

Gold nanoparticles, due to their favourable electrical conductivity, are highly preferred in the detection of biomolecules such as glucose because they are boosters of electrochemical ability. Besides, they always enhance the oxidation of glucose. Hence, they are used as dopants because they help in enhancing an effective transfer of electrons [126, 127]. Interaction of carbon quantum dots (CQDs) with gold AuNPs has been reported to bring about greater electrical conductivity of CQDs, leading to increase in current peak of cyclic voltammetry in biosensors containing CQD-Au nanocomposites [128].

Performance of composite material prepared from gold nanoparticles, carbon nanotubes (CNT), and glassy carbon electrode (GCE) was synergistically exploited in sensing of the electro-oxidation of glucose. The study was carried out using both experimental and theoretical techniques, where DFT and GAUSSIAN 03 W were employed for calculation and optimization, respectively. Three different modified composites were used for the detection of glucose, namely CNT/GCE, Au-CNTs/GCE, and AuNPs/GCE. It was reported that the modified electrodes (prepared from the composites) exhibited remarkable sensitivity towards the detection of glucose [129].

Leveraging on the electron-transfer tendency of gold, electro-oxidation of glucose into gluconic acid has been reported by Tsao and Yang [130] to be enhanced by gold nanoparticles. The gluconic acid produced could easily be detected by matrix-free Laser Desorption/Ionisation mass spectrometry [LDI-MS]. The setup was designed by grafting gold nanoparticles onto a nanostructured silicon surface and was effective in the analysis of glucose present in urine samples. The effect of the fabrication conditions was also examined [130]. The technique leveraged more on mass spectrometry. However, to ensure greater efficiency, accuracy, sensitivity and specificity in electrodes during electrochemical detection of glucose, a new system was developed namely electrochemical and surface assisted laser desorption/ionisation mass spectrometry (EC-SALDI-MS) approach. This bimodal configuration technique (designed using boron-doped carbon nanowalls) takes advantage of the synergy between non-enzymatic electrochemical (EC) and surface-assisted laser desorption/ionisation mass spectrometry. With this technique, a more precise non-enzymatic detection of glucose can be carried out because of its combination of both mass spectrometry and electrochemical applications [126].

Nanocomplex (AuNPs-PVP-GOx) containing AuNPs, polyvinylpyrrolidone (PVP) and glucose oxidase (GOx) was synthesised and found to exhibit sensitivity and selectivity towards D-glucose. The AuNPs, which was synthesised via the Turkevich method, was functionalised using PVP. This functionalization made it possible for AuNPs to effectively bind with GOx, the most used biomolecule in the oxidation and quantification of D-glucose, which was carried out through colorimetric technique. It was found that the amount of peroxide (H_2_O_2_) and triiodide (I_3_^−^) was determined by the concentration of glucose. Thus the biosensor is not only sensitive, its detection performance can also be quantified [131]. The reaction scheme for the formation hydrogen peroxide and triiodide ion is shown in Eqs. (1) and (2), respectively [131].


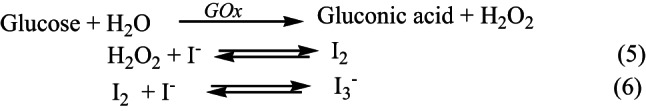


**Fig. 6:**
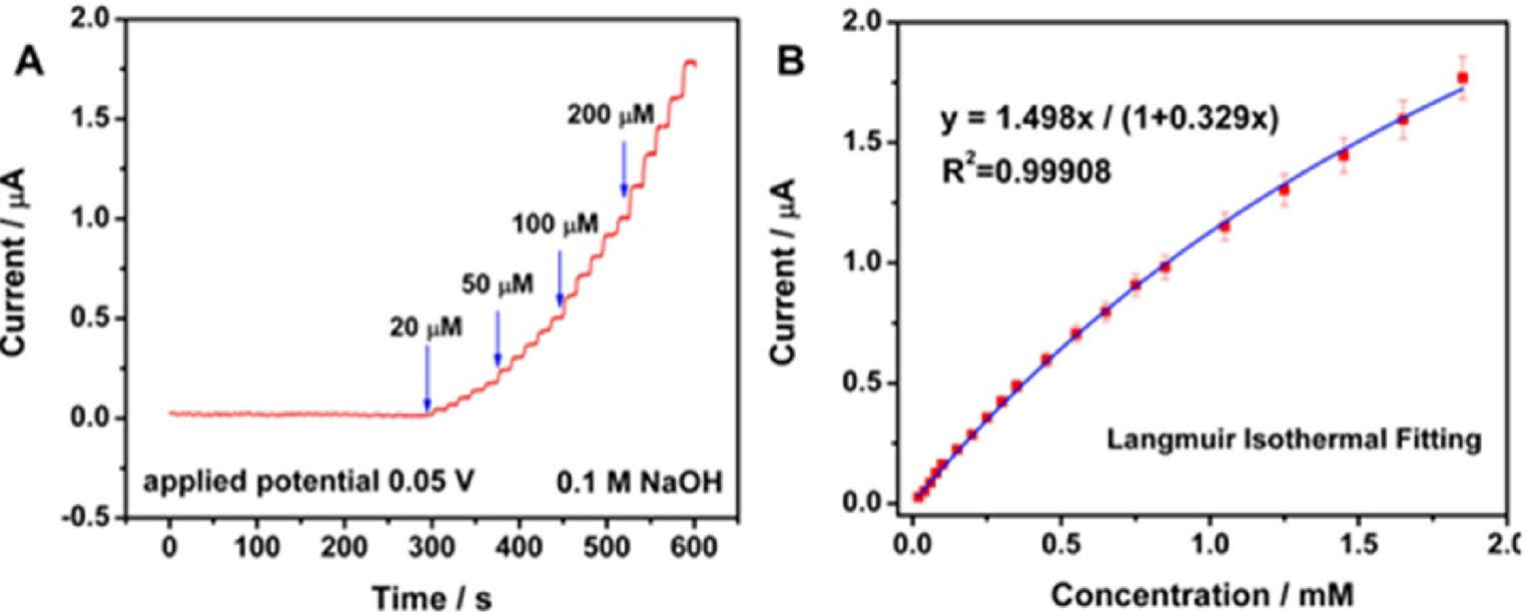
**a** the time interval for the stepwise amperometry response as a function of concentration of glucose injected,** b** the corresponding calibration curve of the current against concentration of glucose [132]

Non-enzymatic detection of glucose was also reported by Dong et al., using heterogeneous Iridium Oxide/Gold Nanoclusters (IrO_2_-Au NCs). The electrochemical sensor produced a sensitivity of 21.20 µA mM^− 1^ cm^− 2^, with a detection limit of 2.93 µM. The IrO_2_-Au nanocomposites prepared was used for modification of glassy carbon electrode employed in the electrochemical measurement. By principle, the oxidation-reduction ability of gold and iridium oxide electrodes in 0.1 M solution of NaOH was exploited. The sensor gave amperometric responses as the concentration of glucose increases/decreases within short intervals (such as 5 s). One advantage of this type of sensor is the moderate selectiveness for glucose in the presence of other compounds which may obstruct accurate reading of glucose concentration in the system to be examined [132]. However, the sensor was reported to be pH sensitive. Figure 6 shows the time taken for amperometric response (current/µA) of the sensor to different concentrations of glucose.

The report of Dong et al., [132] agreed with that obtained from the study carried out by Gao et al., [133], where the selectivity of core-shell gold-nickel nanostructures (Au@Ni/C) towards glucose was investigated. This non-enzymatic glucose sensor is stable, and it also exhibited short detection time (3 s) for detecting 0.5 mmol/L to 10 mmol/L while the estimated sensitivity and detection limit of the sensor were observed to be 23.17 µA cm^− 2^ mM^− 1^ and 0.0157 mM, respectively in 0.1 M NaOH. The amperometric response of the Au@Ni/C sensor was also found to be a function of oxidation of glucose. The performance of this sensor was quite peculiar when compared with other Au-based and Nickel-based sensors it exhibits improved sensitivity and selectivity towards glucose at lower operating voltage. It also displayed high stability against the presence of chloride ion in the system [133].

In a recent development, a subcutaneous method of glucose sensing was reported by Demurtas et al., [134]. The devise involves the use of the model of nanoporous gold (NPG) electrode on Kapton^®^ support, where the electrodes were initially modified with a layer made up of glucose oxidase and Os(2,2′-bipyridine)2Cl⋅PVI (Os(bpy)2Cl PVI) with poly (ethylene glycol) diglycidyl ether Mn 500 (PEDGE-500) as crosslinker. The linear detection range was found to be from 1 to 4 nM, while the sensitivity was 28.6 ± 2.1 µA cm^-2^ mM^-1^. However, both the linear detection range and sensitivity of the electrode reduced to lesser values of 1 to 2.5 mM and 14.6 ± 3.3 µA cm^-2^ mM^-1^, respectively, in artificial plasma. This reduction may likely be attributed to the effect of pH and/or interference from electroactive species present in the plasma. The results obtained were far greater compared to the sensitivity displayed by the glucose sensor (designed using a hybrid of graphene and polyethyleneimine-gold nanoparticles) was 9.3 µA cm^-2^ mM^-1^ [134, 135].

AuNPs deposited on carbon nanotubes were also reported to be active towards the electrochemical detection of glucose in the body. The non-enzymatic glucose biosensor was fabricated by deposition of gold nanoparticles (prepared by reaction of HAuCl_4_.3H_2_O and NaBH_4_, having the average diameter of 7.5 nm) the glassy carbon and screen-printed electrodes. The sensitivity of the biosensor was reported to be 2.77 ± 0.14 µA/mM, while the detection limit was 4.1 µM. However, high tendency of gold nanoparticles to agglomerate led to poor stability; thus causing poor performance after three days [127]. Interestingly, the problem of agglomeration can be solved by exposing the setup to air. This enhances the dispersion of gold nanoparticles and eventually facilitates better stability, performance and reuse.

Ability of gold nanoparticles to enhance transfer of electrons between the electroactive center of glucose oxidase (GOx) and electrode has also been exploited in the detection of glucose up to the concentration of 18 mM. The use of a biosensor based made from Au/MXene nanocomposite was reported to be effective for enzymatic detection of glucose. In the amperometric investigation, sensitivity of 4.2 µA mM^-1^ cm^-2^ and LOD of 5.9 µM were observed. The biosensor was also proven to be stable, reusable and reproducible [136]. Gold/carbon-based materials have potential to serve as effective analytical instruments for detection and identification of wide range of biomolecules in nature.

### Thiols detection using gold/carbon-based materials

Gold-functionalized carbon-based materials have garnered significant attention in biosensing due to their unique electrical, chemical, and surface properties [137]. One important area of application is the detection of thiols also known as mercaptans or sulfhydryl compounds, which are compounds that contain sulphur bonded to a hydrogen atom [138]. These compounds include key biological markers for oxidative stress such as glutathione, cysteine and homocysteine [139]. A hybrid material consisting of gold-platinum nanoparticles electrodeposited onto glassy carbon electrode surface modified with ammonium carbamate was developed for the electrochemical detection of glutathione (GSH) [140]. The resulting Am-Au@Pt/GCE nanocomposite exhibited excellent electrocatalytic activity and stability toward GSH oxidation. The sensor displayed linear response in the GSH concentration range of 0.1–11 µM and achieved a low detection limit (LOD) of 0.051 µM. In another study a gold-functionalized carbon-based sensor was fabricated by decorating a nanoporous poly(3,4)ethylene dioxythiophene film with gold nanoparticles on a glassy carbon electrode. The resulting AuNP-PEDOT/GCE sensor provided a highly conductive and porous matrix for efficient electron transfer and thiol binding via Au-S interactions. The sensor demonstrated a sensitive and selective response to GSH with a detection limit of 0.173 µM and a linear range of 0.5–10 µM [141]. Moreover, gold nanodendrites decorated on flexible carbon fiber electrodes were synthesized via an eco-friendly one-step electrochemical deposition method offering an ultrasensitive platform for detecting L-cysteine. The fabricated sensor exhibited an exceptionally low detection limit of 0.16 nM, a wide linear range of 100–3000 nM and a high sensitivity of approximately 50.2 µA µM^− 1^ cm^− 2^, along with excellent selectivity and rapid response times (3 s) [142]. Other examples of the gold/carbon materials that have been used for detecting thiols are shown in Table 3.

**Table 3:** Gold functionalized carbon-based materials for thiol detections

Gold functionalized carbon-based material	Thiol detected	Linear response & detection limit	References
Nafion-modified carbon paste electrode with electrodeposited AuNPs	Glutathione (GSH)	1.0 × 10⁻⁸–1.6 × 10⁻⁵ M and 2.0 × 10⁻⁵–2.0 × 10⁻⁴ M; LODs: 3.9 × 10⁻⁹ M and 8.2 × 10⁻⁸ M	[143]
FRET-based fluorescence probe using N, S dual-doped carbon dots and AuNPs	Glutathione (GSH)	3.8–415.1 µM; LOD: 0.21 µM	[144].
Gold–carbon composite electrode (AuCE)	Glutathione (GSH)	0.5 × 10⁻⁸–4.2 × 10⁻⁸ mol/dm³; LOD: 2.5 × 10⁻⁹ mol/dm³	[145]
FRET sensor using N-doped carbon quantum dots and gold nanorods (CQDs-AuNRs)	Glutathione (GSH)	Not explicitly stated; fluorescence recovery used for detection	[146]
FA-functionalized rGO loaded with BSA-stabilized Au nanoclusters (FA-rGO-BSA/AuNCs)	Glut athione (GSH)	0–1.75 µM; LOD: 0.1 µM	[147]
Laser-ablated AuNP-decorated MWCNT (LAMWCNT–Au/GCE)	Glutathione (GSH)	0.1–9 µM; LOD: 0.93 µM	[148]
Chitosan-stabilized AuNPs (CS-AuNPs)	Glutathione (GSH)	0.5–50 µM; LOD: 0.21 µM	[149]
Graphitic carbon nitride-supported Au nanoparticles (Au–CN)	L-Cysteine	LOD: 0.48 µM; Sensitivity: 5.8 µA µM⁻¹ cm⁻²	[150]
Gold nanodendrites decorated flexible carbon fiber electrode (Au NDs@FCF)	L-Cysteine	100–3000 nM; LOD: 0.16 nM; Sensitivity: ~50.2 µA µM⁻¹ cm⁻²	[151]
Gold nanostar@graphene quantum dot (AuNS@GQD) composite	Cysteine	Colorimetric detection with LOD of 0.35 nM	[152]
Au nanoparticles decorated holey g-C_3_N_4_ (Au@hg-C_3_N_4_)	Cysteine	High sensitivity with dual-mode SERS and SALDI-MS; selective detection of L- and D-cysteine enantiomers in human serum	[153]
Au nanoparticles on hollow carbon spheres (AuNPs@HCS)	Homocysteine	Detection limit: 4.44 nM; wide detection range; high selectivity and good recovery in serum	[154]
Au nanoparticles/graphene sponge (Au NPs/GS)	Homocysteine	Linear range: 1–100 µM; LOD: 1 µM	[155]
Au NPs/carboxylated MWCNTs (Au NPs/cMWCNTs/GCE)	Homocysteine	Wide linear range; LOD: 28.9 nM	[156]

### Peroxide detection using gold/carbon-based materials

AuNPs have been recently applied as sensors for detecting different molecules, including peroxides. Their unique optical and electrochemical properties make them ideal candidates for sensitive and specific detection methods. Colorimetric hydrogen peroxide (H_2_O_2_) detection by AuNPs occurs through a noticeable color change from red or blue/purple that AuNPs undergo when exposed to peroxides. AuNPs may aggregate, resulting in a visible color shift, which can be quantitatively analyzed using UV/Vis spectroscopy. The surface of AuNPs has been functionalized with distinctive ligands that selectively bind to peroxides, enhancing their sensitivity and detection.

Zhang and coworkers [157] applied AuNPs-GO to sense H_2_O_2_ in spiked and naturally contaminated samples of sterilized milk, apple juices, watermelon juice, coconut milk, and mango juice. The authors reported that the AuNP-GO biosensor exhibited good electrocatalytic efficiency towards the reduction of H_2_O_2_ and that the disposable biosensor could offer great potential for rapid, cost-effective and on-field analysis of H_2_O_2_ in foodstuffs. Berbec et al., [158] fabricated a novel ERGO/AuNPs/GC electrode for the detection of H_2_O_2_. The electrode exhibits high selectivity and low detection limits. The authors observed that the incorporation of the ERGO layer on the AuNPs/GC electrode significantly improved the sensitivity of AuNPs and facilitated the adsorption of H_2_O_2_. In a recent publication, Pupel et al., [159] combined graphene oxide, reduced graphene oxide (ERGO) with gold nanoparticles for electrocatalytic reduction of H_2_O_2_. The authors studied the electrocatalytic efficiency of different shapes of the AuNPs with similar sizes on the electrocatalytic properties. The interaction between AuNPs, GO and ERGO affected the GO structure, and the production of higher sensitivity electrodes was reported.

### Detection of virus, bacteria and other microbes using gold/carbon-based materials

Carbon-based materials (CBMs) such as CQDs, graphene quantum dots (GQDs), CNTs, graphene, and their derivatives have gained widespread applications as nanosensors, especially in the biosensing of microorganisms. This is due to their unique structural and dimensional properties, high surface area, biocompatible, and low toxicity [160]. In biosensing applications, various functionalization approaches have been used to improve the performance of CBMs in the detection of microbial analytes in various samples [161–164]. For instance, studies have reported the modification of the surface functional group of graphene oxide (GO) which has a limited amount of carboxylic acid group (–COOH). The increase in the COOH moieties on GO promotes its conjugation and improves its versatility in biosensing applications [165]. However, in recent times studies have further explored the use of transition metals, rare earth metals, and metals for the functionalization of carbon-based materials. This approach leverages the unique properties of these metals to enhance biosensor performance [166].

**Fig. 7:**
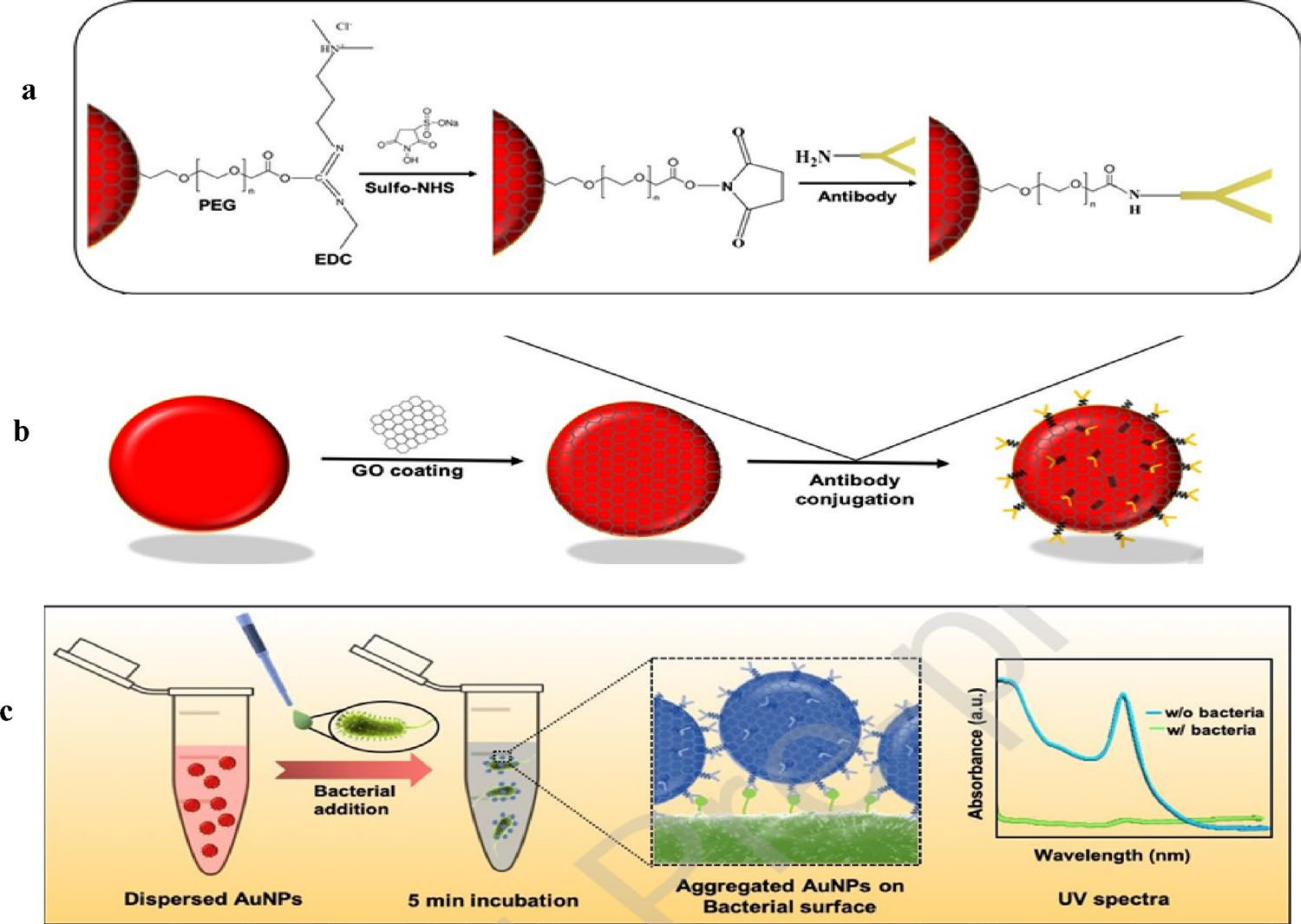
Synthesis of antibody-conjugated PEG-GO-AuNPs (**a**-**b**); Schematic illustration for the mechanism of colorimetric change of nanoprobe upon bacterial addition (**c**). *Image adapted from* [169] with permission from Elsevier

Among the myriads of metals studied more focus has been on transition metals with an emphasis on gold nanoparticles (AuNPs) due to their unique optical and electrochemical properties [167]. The functionalization of CBMs with gold nanoparticles (AuNPs) creates composites with highly sensitive, selective, and robust biosensors. The use of gold functionalization also plays a key in biocompatibility, stability and surface plasmon resonance (SPR) properties which are vital in optical sensing. This also facilitates electron transfer in the fabricated electrochemical sensors thereby enhancing signal amplification and immobilization of biomolecules [168]. An instance was reported by Kaushal et al., [169] using antibody-assisted graphene oxide coated with gold nanoparticles for rapid bacterial detection. The study covalently linked the antibody (Anti- *Salmonella* and Anti *Escherichia. coli*) on pegylated GO-coated AuNPs (PEG-GO-AuNPs). The study revealed that fabricated composite deploys the unique optical and plasmonic properties of gold used in coating the graphene oxide as a hybrid antibody biosensor. It reported appreciable features of fast, specific, and higher sensitivity for the detection of foodborne bacteria while grafting polyethylene glycol (PEG) onto the GO-coated AuNPs (Fig. 7). Using the colorimetric detection approach, the study reported an appreciable of 10^3^ and 10^2^ CFU (Colony Forming Unit), respectively by incubating the probe for 5 min with *Escherichia coli* and *Salmonella typhimurium*. It also noted rapid optical detection by antibody-functionalized hybrid nanoparticle-based biosensors. Also, Rashid et al., developed an electrochemical sensor based on gold nanoparticles-functionalized reduced graphene oxide (rGO) screen-printed electrodes for the detection of pyocyanin biomarkers in *Pseudomonas aeruginosa* infection [170]. The developed electrochemical pyocyanin sensor (AuNPs/rGO) exhibited a good linear range for the determination of pyocyanin in phosphate-buffered saline (PBS), human saliva and urine at a clinically relevant concentration range of 1–100 µM, achieving a detection limit of 0.27 µM, 1.34 µM, and 2.3 µM, )

The impacts of the gold functionalization in the composites were equally reported by Elkhawaga et al., [171]. The study revealed the modification of indium tin oxide (ITO) electrode with polyaniline and the addition of AuNPs functionalization improved the electrochemical signal of pyocyanin generated four times compared to the conventional biosensor. This improved detection efficiency is a reflection of the synergism of the CBMs and Au which promotes the availability of active surface area of the electrode. The presence of these metals also facilitates desirable electron transfer rates as reported by Cernat et al., [172]. The functionalization of various carbon-based materials as biosensors is reported in Table 4 with their respective LOD and their targeted microbial analyte. Pourmadadi et al., [173] reported that decorating reduced graphene oxide with gold nanoparticles facilitated rapid immobilization of aptamers on the electrode surface and enhanced the electrochemical conductivity of the graphene sheets during its selective detection of lipopolysaccharides from Escherichia coli bacteria. Mogha et al., [174] also developed a highly sensitive and selective biosensor for the detection of Mycobacterium tuberculosis via the use of gold nanoparticles immobilized over reduced graphene oxide nanoribbons (RGONRs). The study uses the composite for selective detection of single-strand DNA (ssDNA) of Mycobacterium tuberculosis. The ssDNA/Au/RGONR bioelectrode adopted cyclic voltammetry and chronoamperometric methods for the detection of this microorganism with a report of excellent specificity (92%) in the analyte DNA and high detection efficiency of 0.1 fM. The research added that the functionalization of the CBM with Au as bioelectrode exhibited better signal amplification and electrochemical response as compared to bare Au and RGONR electrodes.

Furthermore, literature has previously noted that direct immobilization of biomolecules onto CNTs or graphene oxide (GO) has been proved unstable. Therefore frequently applied washing steps in biosensor fabrication can readily remove the molecules that impair their stability and exhibit poor reliability/repeatability and non-specificity of the sensor [175, 176]. Khalil et al., [177] also noted that individual sheets of graphene tend to irreversibly self-agglomerate due to van der Waals and stacking interactions, which reduces their electrochemical properties.

**Table 4:** Au-functionalized carbon-based composites and their microbial detection

Au-functionalized Carbon-based composites	Techniques	LOD	Analyte	References
PEG-GO-AuNPs	Colorimetric	10^3^ and 10^2^ CFU	*Salmonella and E. coli in food*	[169]
AuNPs/rGO	Electrochemical	0.27–2.4 µM	*Pseudomonas aeruginosa*	[170]
ssDNA/Au/RGONR	Chronoamperometric methods	0.1 fM	*Mycobacterium tuberculosis*	[174]
ERGO–AuNP	Electrochemical immunosensor	1.19 × 10^2^ CFU/mL	*Enterobacter sakazakii* (*E. sakazakii in food*	[178]
rGOssDNA-AuNPs	Electrochemical impedance spectroscopy (EIS)	0 cfu mL^− 1^	*Staphylococcus aureus*	[179]
Au/MNP-CNTs	Electrochemical	8.4 pM and 8.8 pM	Influenza and norovirus	[180]
PPy/AuNP/MWCNT/Chitosan	Electrochemical immunosensor	∼30 cfu/mL	*Escherichia coli in food*	[181]
AuNPs and NCNO	Electrochemical	3 CFU/mL	*Staphylococcus aureus*	[182]
HBsAb/AgNPs/AuNPs-CNT/SPCE	Differential Pulse Voltammetry	0.86 ng mL^− 1^	Hepatitis B surface antigen	[183]
SPE/EGr-Au	Electrochemical	0.33 µmol L^− 1^	*Pseudomonas* aeruginosa	[184]
Au@SCX8-RGO-TB	Electrochemical biosensor	200 copies/mL	SARS-CoV-2	[185]
GO-AuNPs	Impedimetry/ Immunosensor	1.2 × 10^2^ cfu mL^− 1^	*Cronobacter sakazakii*	[178]
Au-GO paper	Impedimetry/ Immunosensor	1.5 102 cfu mL^− 1^	*E. coli* O157:H7	[186]
GCE-GO-Au Nps	Impedimetry/Aptasensor	3.0 × 10^0^ cfu mL^− 1^	*Salmonella*	[187]
GOx-Thi-Au@SiO_2_	Amperometry/Genosensor	0.01 nM	*E. coli* O157:H7	[188]
Pyrrole/gold MWCNTs/chitosan	Amperometry/ Immunosensor	3.0 × 10^− 1^ cfu mL^− 1^	*E. coli* O157:H7	[189]
AuNPs/PAMAM-MWCNT-Chi/GCE	Electrochemical impedance immunosensor	5.0 × 10^2^ cfu mL^− 1^	*Salmonella*	[190]

*CFU* colony forming unit, *GO* graphene oxides, *Au* gold nanoparticles, *PEG* polyethylene glycol, *GCE* glassy carbon electrode, *ssDNA* single-stranded DNA, *MNP* magnetic nanoparticles, *CNP* carbon nanotubes, *PPy* phosphonium pyrophosphate, *MWCNT* multi-walled carbon nanotubes, *HBsAb* Hepatitis B surface antibody, *PANAM* polyampholytic

Moreover, concerns have been raised about the surface energetics of nanoscale materials, as they exhibit a very high surface area-to-volume ratio. These challenges highlight the need for surface functionalization with agents such as metals, metal oxides, surfactants and small organic molecules [161, 191, 192]. Among these functionalizing agents, especially noble metals, gold acts as a nano-spacer and conductor in the composite. This increases the graphene interlayer distance to minimize the agglomeration, making both faces accessible and improving the electrical conductivity [177, 193, 194]. Thus, the impacts of Au coupled with CBM present a composite biosensor with remarkable attributes of higher chemical stability and biocompatibility. This makes it a model component for rapid detection and identification of microorganisms [176]. In addition, Hu et al., [178] added that the intercalation of AuNPs into ERGO sheets improved the detection and conductivity of the composite used as an immunosensor based on the synergistic effect of Au coupled with electrochemically reduced graphene oxide (ERGO) for the detection of *E. sakazakii* in food. This was accomplished via the superior electron transporting property of rGO and the surface properties of AuNP which enhance the aptasensor detection capability of the sensor for *Staphylococcus aureus* [179]. Similarly, Lee et al., [180] reported the successful accomplishment of high sensitivity and selectivity detection of influenza and norovirus DNA using Gold (Au)/iron-oxide magnetic NP-decorated CNTs (Au/MNP-CNT). From the TEM studies the AuNPs were grown using only the reducing agent (oxidizable magnetic nanoparticle) without a surface stabilizer and coupled with CNT. These hybrid materials account for improved electrical conductivity for the detection of the target virus DNA. Also, the provision of a reliable means of quantification of the bacteria (*E.coli*) brought about the development of a unique hybrid PPy/AuNP/MWCNT@Chitosan [181]. The study identified that the inclusion of Polypyrrole (PPy) is based on its notable environmental stability and excellent electrical conductivity which is adduced to conducting polymers [195].

However, the cyclic voltammetry characterization of the composites indicates the highest observed peaks at the AuNPs doped curves which indicates the impacts of Au in mix promotes significant electron transport thereby improving the conductivity of the fabricated biosensor. This also enhanced low LOD as presented in the Table 4 while the functionalization improved the selectivity of the biosensor while providing affinity between the antibodies and the bacteria. This was also similar to fabrication of composites comprising of nitrogen-doped carbon nano-onions (NCNO), gold nanoparticles (AuNPs) and thiol-terminated, via self-assembled on the electrode surface through the covalent modification of thiol groups with AuNPs. The processes resulted in the fabrication of aptamer-modified, screen-printed carbon electrodes for the detection of *S. aureus* [182]. Also, Upan et al., [183] investigated the built screen-printed carbon electrode (SPCE) was modified with a carbon nanotube decorated with gold nanoparticles (AuNPs-CNT) and silver nanoparticles AgNPs. The application of the composites was noted with features of high selectivity and reproducibility. The study also added that the functionalization of the carbon-based materials with the noble metals especially gold provides the blend with appreciable biocompatibility and large surface area. This enhances the dense immobilization of hepatitis B surface antibody (HBsAb) on the electrode, which helps to improve the signal. This action was similarly reported by Gandouzi et al., [184] when graphene was functionalized with gold nanoparticles.

The modification of Screen-Printed Carbon Electrode (SPCE) with AgNPs and AuNPs coupled with CNT was analyzed using Electrochemical impedance spectroscopy (EIS) to observe the electrode performance vis-à-vis the modification. The study holds that after modification of SPCE with AgNPs and AuNPs coupled with CNT, there was a significant improvement in the electron transfer rate of the redox probe at the surface of the electrode [183]. This agrees with the excellent electric conductivity and effective electron transfer at the surface resulting in the decrease of the repulsive forces occurring between electrode/electrolyte [184]. The reported underscored electrochemically generated graphene composite that was chemically functionalized with AuNPs using a layer-by-layer technique. The combination of these two nanostructures in the composite material created a synergistic effect, resulting in significantly higher detection efficiency with lower LOD for microbial analyte.

## Conclusion and future perspective

The review highlights the remarkable potential of gold-functionalized carbon-based composites in advancing biosensing technologies. The synergistic integration of AuNPs with carbon-based materials significantly enhances electrical properties, surface area, biocompatibility and functional group availability enabling highly sensitive and selective detection of wide spectrum of analytes. These hybrid materials have been successfully used to detect microbes, tumor cells, heavy metals, protein enzymes, thiols, peroxide and glucose. However, most of these detection applications have been tested on single analyte and they face reproducibility and stability issues under real world conditions, thereby restricting the scaling up for large industrial scale application. Looking forward future research on gold functionalized carbon-based materials should focus on (1) developing multiplexed biosensors capable of detecting multiple analytes simultaneously, (2) exploring sustainable and cost-effective and scalable functionalization methods without compromising performance and (3) testing these materials on real samples and large-scale field trials to accelerate transition from lab prototype to commercial products. Finally, the use of these gold/carbon-based materials as biosensors for monitoring biomolecular processes, such as enzymatic interactions, antibody/antigen interactions, and DNA interactions should be further explored.

## Data Availability

No datasets were generated or analysed during the current study.
